# Stromal Fibroblasts Mediate Extracellular Matrix Remodeling and Invasion of Scirrhous Gastric Carcinoma Cells

**DOI:** 10.1371/journal.pone.0085485

**Published:** 2014-01-10

**Authors:** Hideki Yamaguchi, Nachi Yoshida, Miho Takanashi, Yuumi Ito, Kiyoko Fukami, Kazuyoshi Yanagihara, Masakazu Yashiro, Ryuichi Sakai

**Affiliations:** 1 Division of Metastasis and Invasion Signaling, National Cancer Center Research Institute, Chuo-ku, Tokyo, Japan; 2 Laboratory of Genome and Biosignal, Tokyo University of Pharmacy and Life Sciences, Hachioji-shi, Tokyo, Japan; 3 Research Center for Innovative Oncology, National Cancer Center Hospital East, Kashiwa-City, Chiba, Japan; 4 Department of Surgical Oncology, Osaka City University Graduate School of Medicine, Abeno-ku, Osaka, Japan; Vanderbilt University Medical Center, United States of America

## Abstract

Scirrhous gastric carcinoma (SGC) has the worst prognosis of all gastric cancers, owing to its rapid expansion by invasion and frequent peritoneal dissemination. Due to the increased proliferation of stromal fibroblasts (SFs) that occurs within SGC lesions and the peritoneal metastatic sites, SFs have been proposed to support the progression of this disease. However, the biological and molecular basis and the pathological role of the intercellular interaction between SGC cells and SFs remain largely unknown. In this study, we investigated the role of SFs in the invasion of the extracellular matrix (ECM) by SGC cells. When SGC cells were cocultured with SFs derived from SGC tissue on three-dimensional (3D) Matrigel, they were attracted together to form large cellular aggregates that invaded within the Matrigel. Time-lapse imaging revealed that this process was associated with extensive contraction and remodeling of the ECM. Immunofluorescence and biochemical analysis showed that SGC cells stimulate phosphorylation of myosin light chain and actomyosin-mediated mechanical remodeling of the ECM by SFs. By utilizing this assay system for inhibitor library screening, we have identified several inhibitors that potently suppress the cooperation between SGC cells and SFs to form the invasive structures. Among them, a Src inhibitor dasatinib impaired the interaction between SGC cells and SFs both in vitro and in vivo and effectively blocked peritoneal dissemination of SGC cells. These results indicate that SFs mediate mechanical remodeling of the ECM by SGC cells, thereby promoting invasion and peritoneal dissemination of SGC.

## Introduction

Recent studies have established the importance of the tumor stroma in cancer progression [1], [2]. Tumor stroma consists of many types of non-cancerous cells and non-cellular components including the extracellular matrix (ECM). Stromal fibroblasts (SFs) are major cellular constituents of tumor stroma and often called cancer-associated fibroblasts (CAFs) [3]. They have been implicated in the malignant behavior of cancers, such as cell proliferation, ECM remodeling, and angiogenesis [4]. Moreover, they often display the phenotypes of myofibroblasts, characterized by the expression of α-smooth muscle actin (αSMA) and strong contractility [5]. These characteristics contribute not only to fibrosis in tumor tissue but also to the remodeling and stiffening of the stromal ECM that are favorable for invasion and metastasis of carcinoma cells [6], [7].

Scirrhous gastric carcinoma (SGC), also known as diffusely infiltrative carcinoma, has a very poor prognosis due to rapid infiltrative invasion and a high incidence of peritoneal dissemination [8], [9]. SGC is associated with extensive stromal fibrosis, resulting in the thickening and hardening of the gastric wall and shrinkage of the stomach. As there is elevated proliferation of SFs in SGC lesions, they have been proposed to support the progression of this disease [10]. In fact, a positive correlation between the presence of SFs and the metastatic potential of gastric cancers has been found [11]. SGC cells induce fibrosis of the peritoneum in peritoneal dissemination, indicating that SFs also play a role here [12]. Recent studies have demonstrated that SFs stimulate migration and invasion of SGC cells [13], [14] and SGC cells reciprocally promote proliferation of gastric fibroblasts [14], [15]. However, the biological and molecular basis and the pathological function of the intercellular interaction between SGC cells and SFs remain largely unknown. In this study, we established the system to visualize and quantify the crosstalk between SFs and SGC cells for achieving the invasive properties and investigated the role of SFs in the invasion and remodeling of the ECM by SGC cells.

## Materials and Methods

### Cell culture

Human gastric cancer cell lines 58As9, HSC-59, HSC-44PE, and 44As3 were described previously [16], [17], and MKN1, MKN7, and MKN74 were obtained from the Health Science Research Resources Bank. 44As3 cells stably expressing tdTomato were generated by retroviral transduction. These cells were maintained in RPMI 1640 medium (Invitrogen) supplemented with 10% FBS and antibiotics at 37°C in a humidified atmosphere containing 5% CO_2_. The orthotopic fibroblast cell lines, CaF37 and CaF38 were established from the tumoral gastric wall of SGC patients who had undergone gastrectomy. The primary gastric tumor was excised under aseptic conditions and minced with forceps and scissors. The tumor pieces were cultivated in DMEM (Nikken) with 10% FBS. After approximately 2 weeks, fibroblasts were collected and transferred to another culture dish. Serial passages were carried out every 4–7 days. The fibroblasts used were 4–10th passage of culture. This study was approved by the Osaka City University ethics committee and written informed consent was obtained from the patients prior to the study.

### Reagents

Fluorescent phalloidin and secondary antibodies, CellTracker, and FluoSpheres polystyrene microspheres (1.0 μm, red fluorescent, 580/605) were purchased from Invitrogen. Matrigel was purchased from BD Biosciences. Inhibitors used were; GM6001, blebbistatin, and PP2 (Merck), dasatinib (Selleck Chemicals), and H1152 and imatinib (Cayman Chemical).

### Immunoblotting

Immunoblotting was performed as described previously [18]. Protein concentration was determined with the BCA protein assay kit (Pierce). Following antibodies were used: αSMA, α-tubulin, and ß-actin (Sigma-Aldrich) at 1∶5000 dilution; myosin light chain 2 (MLC2), phospho-MLC2 (Ser19), phospho-MLC2 (Thr18/Ser19), Src, phospho-Src (Tyr416), and vimentin (Cell Signaling Technology) at 1∶1000 dilution.

### 3D Matrigel invasion assay

Matrigel was placed in 24-well plates (250 µl/well) or on cover slips (12-mm, circular; 40 µl/cover slip) and solidified at 37°C for 1 h. SGC cells (2×10^4^) and SFs (5×10^4^) were plated onto 3D Matrigel and cultured for 2 days. To visualize ECM remodeling, Matrigel was mixed with FluoSpheres polystyrene microspheres at 2×10^3^ beads/ml. To calculate the number of invasive foci, Matrigel-containing plates were scanned and analyzed with the particle counting function of the ImageJ 1.41o software. Cell aggregates larger than 0.05 mm^2^ in area were counted in this analysis. In each experiment, the values of control cells were set to 1 and the relative values of other cells were then calculated accordingly. To calculate the number and areas of cell clusters, cells in six randomly selected fields were imaged with a 10× objective and analyzed with the ImageJ 1.41o software. Invasion depth of invasive foci was determined by confocal microscopy.

### Gelatin remodeling assay

Fluorescent gelatin-coated cover slips were prepared as described previously [19]. Cells were cultured on the gelatin-coated cover slips for 16 h. To quantitate the gelatin remodeling activity, areas where the fluorescent gelatin was detached were calculated in microscopic images using the ImageJ 1.41o software. Ten randomly selected fields were imaged with a 20× objective and analyzed for each experiment. The values of control cells were set to 100, and the relative values of other cells were then calculated accordingly.

### Immunofluorescence and time-lapse microscopy

Immunofluorescence was performed as described previously [19]. Phospho-MLC2 (Ser 19) antibody was used at 1∶50 dilution. FSP1/S100A4 antibody (Millipore) was used at 1∶200 dilution. Samples were observed with an Olympus IX81-ZDC-DSU microscope equipped with a cooled CCD camera (ORCA-ER, Hamamatsu), and the imaging system was driven by MetaMorph software (Universal Imaging). For Time-lapse microscopy, 44As3 cells (1×10^4^) and CaF37 cells (2.5×10^4^) were plated onto 3D Matrigel (120 µl/well) solidified in a multi-well glass bottom dish (Matsunami). To quantitate ECM remodeling, digital images were converted using ImageJ 1.41o and the movement of microbeads was analyzed. Total distance moved during the time-lapse imaging was calculated for each microbead and normalized by the average value in 44As3 cells. The total and net migration distances of cells before the formation of invasive foci were determined from the time-lapse movies with the manual tracking function of the ImageJ 1.41o software.

### Cell growth assay

Cells were plated onto 96-well plates at 4×10^3^/well and cultured for 2 days in the presence of inhibitors. Cell growth was determined using a Premix WST-1 Cell Proliferation Assay System (Takara) according to manufacturer instructions. Absorbance at 450 nm was measured with an iMark microplate reader (Bio-Rad Laboratories). The average values of cells treated with DMSO were set to 1, and the relative values of other cells were then calculated accordingly.

### Cytotoxicity assay

Cells were plated onto 96-well plates at 1×10^4^/well and cultured for 1 day in growth medium. Medium was replaced with RPMI1640 containing 1% FBS and cultured for 2 days in the presence of inhibitors. Cytotoxicity was then determined using a LDH Cytotoxicity Detection Kit (Takara) according to manufacturer instructions. Absorbance at 490 nm was measured with an iMark microplate reader (Bio-Rad Laboratories). The average values of cells treated with 1% Triton X-100 were set to 1, and the relative values of other cells were then calculated accordingly.

### Inhibitor screening

SCADS inhibitor kit was kindly provided by Screening Committee of Anticancer Drugs, Japan. Matrigel was placed in µ-Slide Angiogenesis (ibidi, 10 µl/well) and solidified at 37°C for 1 h. SGC cells (2×10^3^) and SFs (5×10^3^) labeled with CellTracker were cultured in each well in the presence of the inhibitor library (10 µM) for 2 days. The µ-Slide chambers were scanned and the number of invasive foci was calculated. The cells were also observed by fluorescent microscopy and the formation of invasive foci was evaluated.

### Peritoneal dissemination assay

44As3 (5×10^5^) cells were inoculated intraperitoneally into 6-week-old BALB/c nude mice purchased from CLEA Japan (Tokyo, Japan). Dasatinib (50 mg/kg) were administrated by intraperitoneal injection, thrice a week, starting at a day after the inoculation. At 10 days after inoculation, the mice were sacrificed and dissected. Peritoneal dissemination, liver metastasis, and ascites formation were examined. The number of mesentery nodules larger than 1 mm in diameter was also determined. The mesentery nodules were paraffin-embedded, sectioned, and subjected to hematoxylin and eosin staining and immunohistochemistry for histological analysis. For immunofluorescence, mesenteries bearing tdTomato-labeled 44As3 tumor nodules were isolated at 3 days after inoculation. These experiments were approved by the Committee for Ethics of Animal Experimentation and conducted in accordance with the guidelines for Animal Experiments in the National Cancer Center.

### Statistical analysis

Data are representative of at least 3 independent experiments. Statistical analysis was performed using Student's *t*-tests, ANOVA with Tukey's test, or Mann-Whitney test.

## Results

### SGC cells and SFs form invasive foci on 3D Matrigel

**Figure 1:**
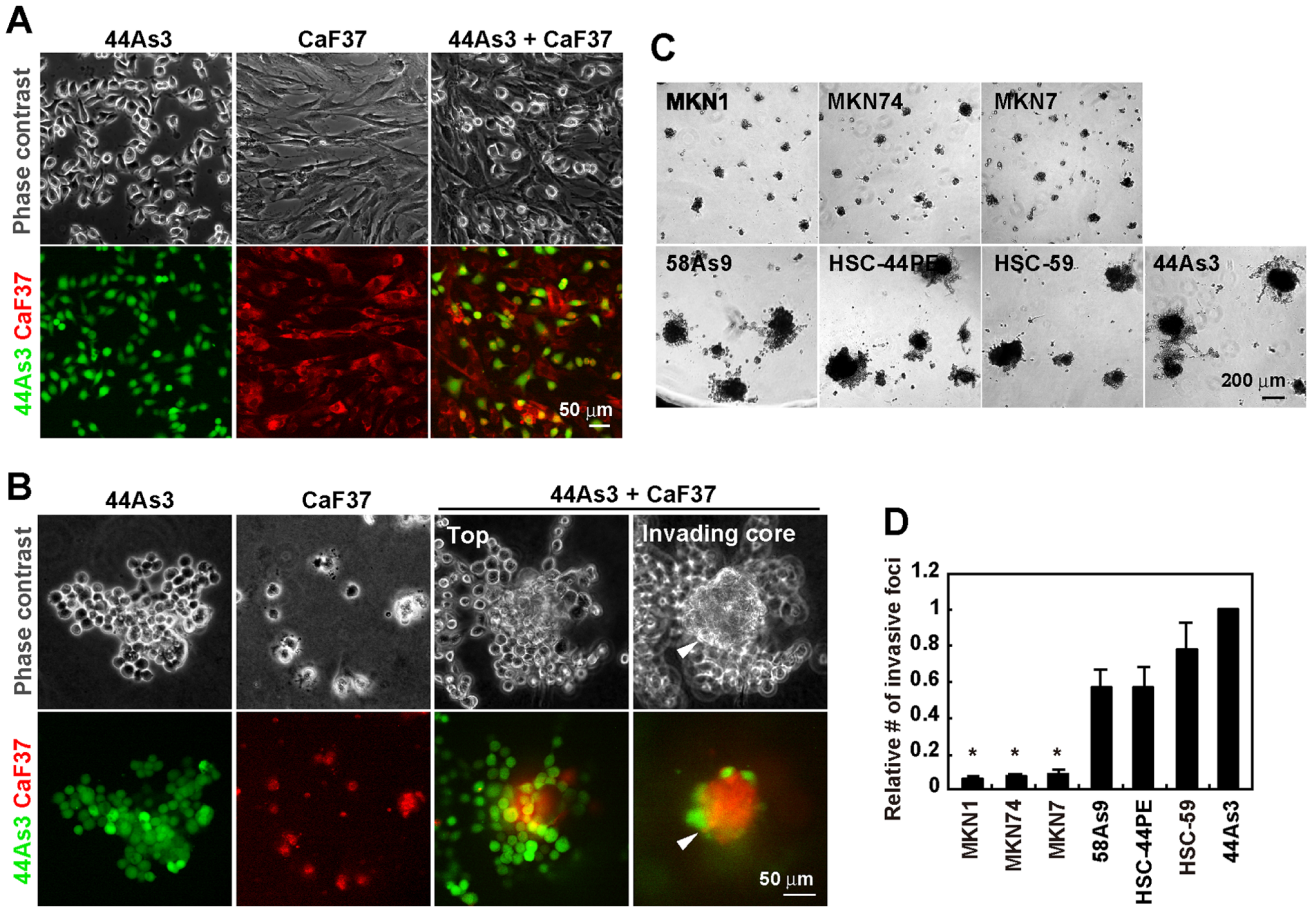
Formation of invasive foci by SGC cells and SFs on 3D Matrigel. A and B, 44As3 and CaF37 cells were labeled with CellTracker and cultured individually or together on either 2D (A) or 3D (B) Matrigel for 2 days. C, Non-SGC gastric cancer cell lines (MKN7, MKN1, and MKN74) and SGC cell lines (58As9, HSC59, HSC44PE, and 44As3) were cocultured with CaF37 cells on 3D Matrigel for 2 days. D, The relative number of invasive foci formed by CaF37 and indicated gastric cancer cell lines. Bars show mean ± SEM (*n* = 4). *, *p*<0.005 vs SGC cell lines, by ANOVA with Tukey's test.

44As3 SGC cells and CaF37 fibroblasts derived from SGC tissue were labeled with different fluorescent dyes and cultured individually or together, on either Matrigel-coated culture dishes (2D) or the more physiological 3D Matrigel matrices. 44As3 cells cultured on 2D Matrigel were adherent to the substrate but showed less cell-cell adhesions (Fig. 1A). CaF37 cells spread on the substrate and had a spindle morphology. Their morphologies did not significantly change when these cells were cultured together on the 2D substrate. 44As3 cells cultured on 3D Matrigel exhibited rounded morphology and formed flat cell clusters, without showing significant invasion into the matrix (Fig. 1B). CaF37 fibroblasts also showed rounded morphology with small membrane protrusions on 3D Matrigel and formed invasive small cell aggregates reaching as deep as approximately 30 µm in average (Fig. 1B and Fig. S1A). When 44As3 and CaF37 cells were cocultured on 3D Matrigel, they formed large aggregates that consist of a sphere-shaped core and surrounding cells (Fig. 1B). These cellular aggregates invaded as deep as approximately 70 µm in average into the Matrigel, indicating greater invasiveness with this coculture compared to the CaF37 cells alone (Fig. S1A). CaF37 fibroblasts localized almost exclusively in the invading core structures and most of the surrounding and co-invading cells were 44As3 cells (Fig. 1B). We hereafter refer to these invasive cellular aggregates formed by SGC cells and SFs as invasive foci. Addition of conditioned medium from 44As3 cells to CaF37 cells, or vice versa, did not affect the behavior of CaF37 or 44As3 cells (Fig. S1B), suggesting that a direct association between the 2 cell types is important for the formation of invasive foci.

Coculture of 44As3 and CaF37 cells markedly increased the number of invasive foci on 3D Matrigel compared to individual cultures (Fig. S1C and D). Similar results were obtained from 44As3 cells cocultured with CaF38 fibroblasts, also isolated from SGC tissue (Fig. S2A and B). Coculture of non-SGC cell lines MKN7 (well-differentiated tubular adenocarcinoma), MKN1 (adenosquamous carcinoma), and MKN74 (moderately differentiated tubular adenocarcinoma) with CaF37 resulted in formation of sparse and small cell aggregates, with little invasive potential (Fig. 1C and D). In contrast, SGC cell lines 58As9, HSC-44PE, and HSC-59 formed large invasive foci with CaF37 cells, which were comparable to those formed by 44As3 and CaF37 cells (Fig. 1C and D). These observations indicate that the formation of invasive foci is distinctive for SGC cells.

### Formation of invasive foci is associated with extensive remodeling of ECM

**Figure 2:**
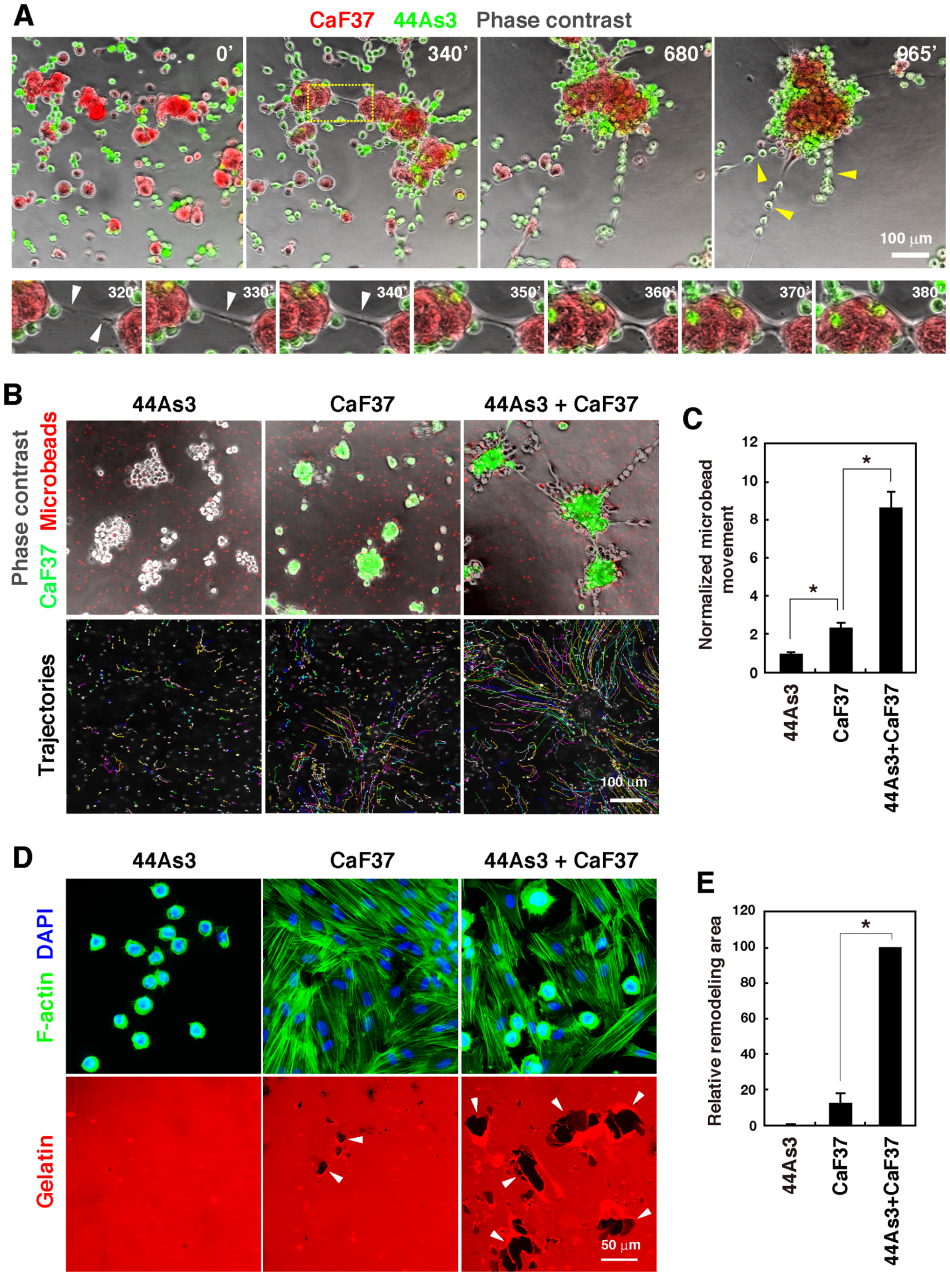
Imaging of the formation of invasive foci and concomitant remodeling of ECM. A, Time-lapse imaging of 44As3 and CaF37 cells cultured on 3D Matrigel. Yellow arrowheads denote chain-like structures formed by 44As3 cells. Lower panels are magnified image sequences of the boxed region. White arrowheads denote cell protrusions of CaF37 cells. B, Movement of the microbeads that were embedded in 3D Matrigel was tracked for 9 h and trajectories of each bead were shown as colored lines. C, Quantification of the movement of microbeads. Bars show mean ± SEM (*n* = 48 beads analyzed). *, *p*<0.0001 by Student's *t*-test. Similar results were obtained in three independent experiments. D, 44As3 and CaF37 cells were cultured on fluorescent gelatin-coated cover slips for 16 h and stained for F-actin and the nucleus (DAPI). Arrowheads denote the black areas where gelatin matrices were removed from cover slips. E, Quantification of the areas of gelatin detachment. Bars show mean ± SEM (*n* = 6). *, *p*<0.0001 by Student's *t*-test.

Time-lapse imaging showed that 44As3 and CaF37 cells exhibited strong migratory phenotypes on 3D Matrigel and gradually gathered to form small clusters, which then coalesced to form large cellular aggregates as time elapsed (Fig. 2A and Video S1). In this example, CaF37 cells within the cell clusters formed long cell protrusions that interacted with each other to attract 2 cell clusters together, resulting in larger cell clusters containing attached 44As3 cells throughout this process. In some cases, 44As3 cells lined up and formed chain-like structures, often on the protrusions of CaF37 cells, which interconnected with small clusters of CaF37 cells. These observations suggest that SGC cells and SFs have strong physical interactions with each other, which contribute to the formation of invasive foci.

Remodeling of ECM was also visualized by tracking the movement of fluorescent microbeads (1.0 µm in diameter) that were seeded in the Matrigel (Fig. 2B and C). When 44As3 cells were cultured alone, the microbeads showed only a slight movement (Video S2). CaF37 cells induced more obvious movement of the microbeads toward each cell cluster (Video S3). When these cells were cultured together, the microbeads showed extensive and long movement along the migratory paths of the cell clusters, finally accumulating around the invasive foci (Video S4). These results indicate that the coculture generated a contraction force during the formation of invasive foci, which resulted in mechanical remodeling and deformation of the ECM.

To characterize further the ECM remodeling activity of SFs, we cultured 44As3 and CaF37 cells on fluorescent gelatin-coated cover slips (Fig. 2D and E). 44As3 cells did not affect the integrity of the gelatin matrix. CaF37 cells induced remodeling of the gelatin matrix as shown by the appearance of black regions. This activity was markedly enhanced when they were cocultured with 44As3 cells. Similar results were obtained when CaF38 cells were used (Fig. S2C and D). This gelatin remodeling activity was not blocked by a broad MMP inhibitor, GM6001 (Fig. S3A and B), while the inhibitor successfully suppressed MMP-dependent degradation of the gelatin matrix by invadopodia in MDA-MB-231 human breast cancer cells (Fig. S3C). Based on these observations, we inferred that this gelatin remodeling was due to mechanical detachment rather than enzymatic degradation.

### Actomyosin-dependent contraction is required for SF-mediated ECM remodeling and invasion of SGC cells

**Figure 3:**
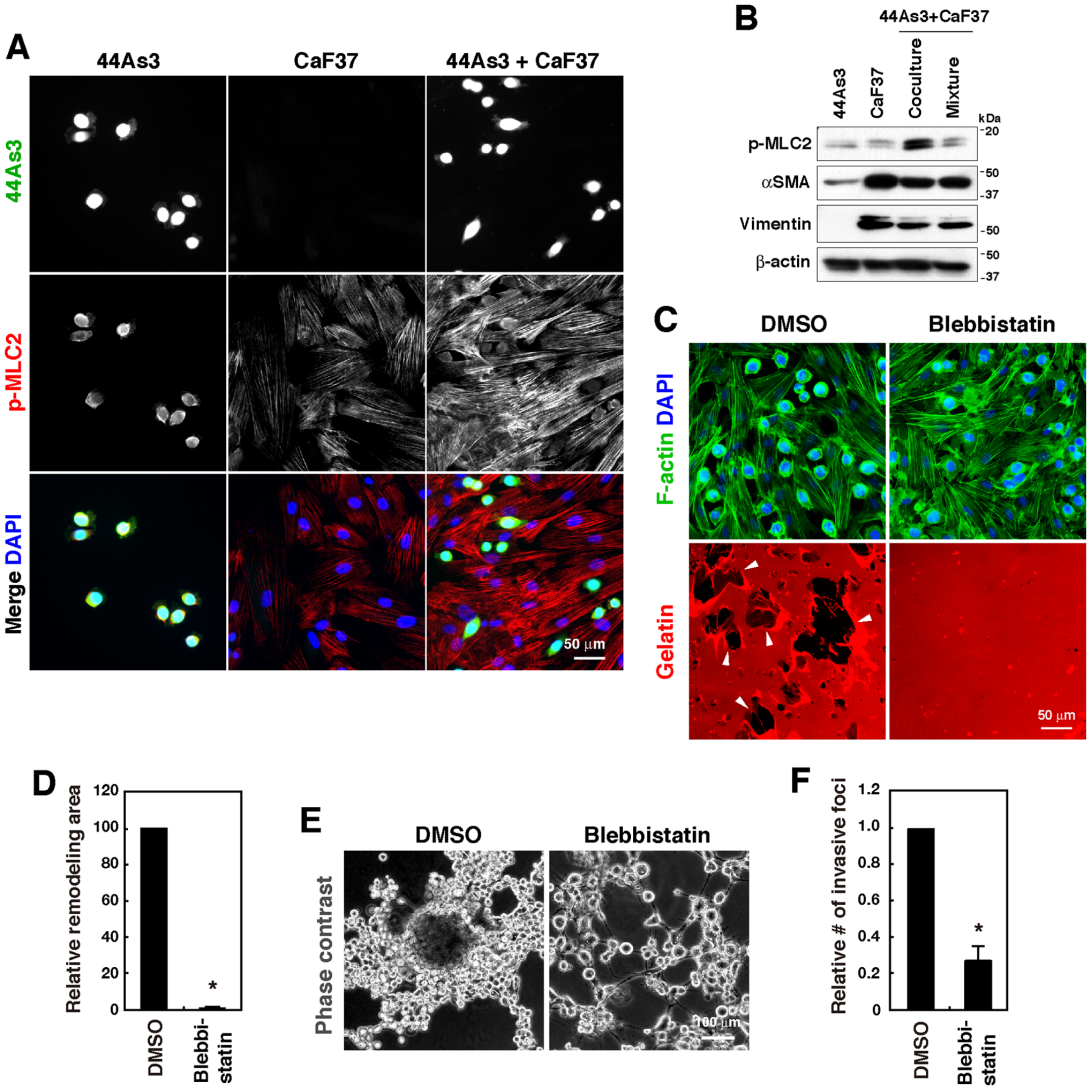
Formation of invasive foci and associated remodeling of ECM require actomyosin contraction. A, 44As3 and CaF37 cells cultured on gelatin-coated cover slips were stained with the antibody against phospho-myosin light chain 2 (p-MLC2) and DAPI. B, Immunoblot analysis of cell lysates prepared from individually cultured or cocultured (coculture) 44As3 and CaF37 cells. A mixture of cell lysates of individually cultured cells was used as a control (mixture). C, 44As3 and CaF37 cells were cultured on fluorescent gelatin in the absence or presence of blebbistatin (10 µM) for 16 h. Arrowheads denote the areas where gelatin matrices were disrupted. D, Quantification of the areas of gelatin disruption. Bars show mean ± SEM (*n* = 4). *, *p*<0.000001 by Student's *t*-test. E, 44As3 and CaF37 cells were cocultured on 3D Matrigel in the absence or presence of blebbistatin (10 µM) for 2 days. F, The relative number of invasive foci was determined. Bars show mean ± SEM (*n* = 4). *, *p*<0.005 by Student's *t*-test.

Mechanical remodeling of the ECM requires cellular force generated by actomyosin-mediated contractility [6], [20]. Immunofluorescence analysis revealed that signals for phospho- myosin light chain 2 (MLC2), an indicator of actomyosin contractility [21], were significantly stronger in CaF37 cells cocultured with 44As3 cells than in those cultured alone (Fig. 3A). Immunoblot analysis also showed that cocultured 44As3 and CaF37 cells have a higher level of MLC2 phosphorylation than in individually cultured cells or in a mixture of these cell lysates (Fig. 3B). Although αSMA and vimentin are strongly expressed in CaF37 cells, the expression levels were unchanged by co-culturing them with 44As3 cells (Fig. 3B). Treatment with a myosin II inhibitor blebbistatin nearly completely blocked disruption of the gelatin matrix and the formation of invasive foci by 44As3 and CaF37 cells (Fig. 3C–F). In contrast, GM6001 treatment did not affect these processes (Fig. S3D and E). These results indicate that actomyosin-mediated contractility is necessary for mechanical remodeling of the ECM during invasion by SGC and SFs.

### Inhibitor library screening identified Rock and Src as critical regulators of the formation of invasive foci

**Figure 4:**
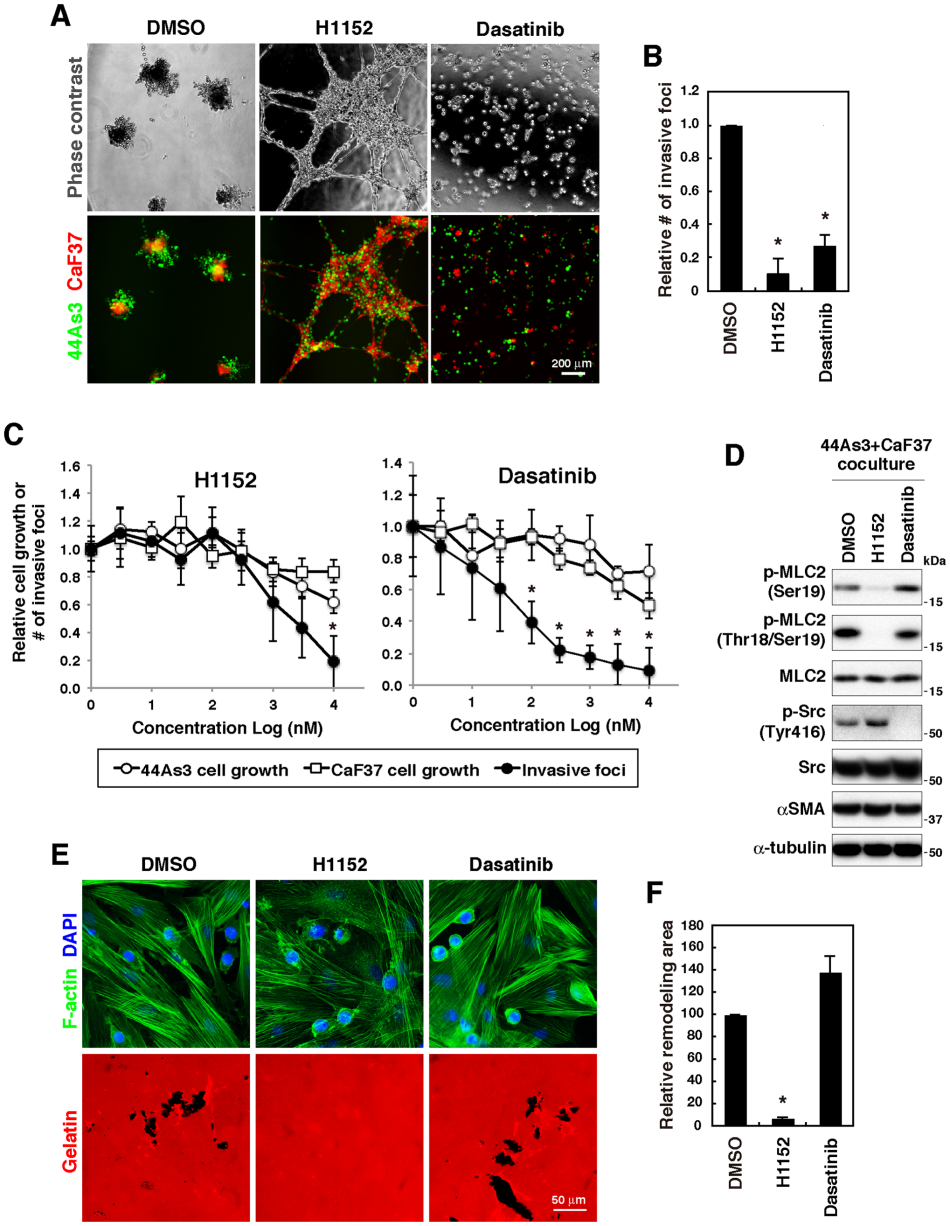
Rock and Src regulate the formation of invasive foci. A, 44As3 and CaF37 cells were plated onto 3D Matrigel in the presence or absence of H1152 (10 µM) or dasatinib (10 µM) for 2 days. B, Quantification of the number of invasive foci. Bars show mean ± SEM (*n* = 3). *, *p*<0.05 by Student's *t*-test. C, Dose response effects of H1152 and dasatinib on cell growth of 44As3 and CaF37 cells and the formation of invasive foci. Bars show mean ± SD (*n* = 8 for cell growth and 3 for invasive foci). *, *p*<0.05 by Student's *t*-test. D, Immunoblot analysis of 44As3 and CaF37 cells that were cocultured and treated with H1152 or dasatinib. E, The effect of H1152 and dasatinib on remodeling of the gelatin matrix. F, Quantification of the areas of gelatin disruption. Bars show mean ± SEM (*n* = 3). *, *p*<0.0005 by Student's *t*-test.

To further gain the mechanistic insights into the formation of invasive foci, inhibitor screening was carried out with the SCADS inhibitor kit, which consists of 292 compounds, including well-known antitumor agents and molecular target drugs. As a result of quantitative analysis, several inhibitors that potently inhibited the formation of invasive foci were identified (Table S1 and Fig. S4). Cytotoxicity against individually cultured 44As3 and CaF37 cells was also determined to exclude compounds with non-specific effects (Table S1). We focused on H1152 and dasatinib, inhibitors for Rock and Src/Abl, respectively, for further analyses, because they have less cytotoxicity and exhibited strong inhibitory effects on the formation of invasive foci (Table S1), as confirmed by detailed fluorescent microscopic and quantitative analyses (Fig. 4A and B). In addition, cells treated with H1152 exhibited unique phenotypes (Fig. 4A): they formed flat and dispersed cell clusters that were interconnected with multicellular extensions. Although both inhibitors moderately suppressed cell growth of 44As3 and CaF37 cells at higher concentrations, they more efficiently blocked the formation of invasive foci (Fig. 4C): IC50 values were approximately 36 nM for dasatinib and 1.7 µM for H1152.

Rock regulates actomyosin contractility by directly phosphorylating MLC [22]. H1152 treatment significantly reduced phosphorylation of MLC2 in co-cultured 44As3 and CaF37 cells (Fig. 4D) and also strongly inhibited disruption gelatin matrix (Fig. 4E and F). Time-lapse observations revealed that although 44As3 and CaF37 cells still migrated and interacted each other by extending cellular protrusions even after treatment with H1152, following compaction of cell clusters was severely impaired as compared with control DMSO-treated cells (Video S5 and S6). This resulted in the formation of loosely packed flat cell clusters that were interconnected with cell protrusions and scarcely invaded into Matrigel.

**Figure 5:**
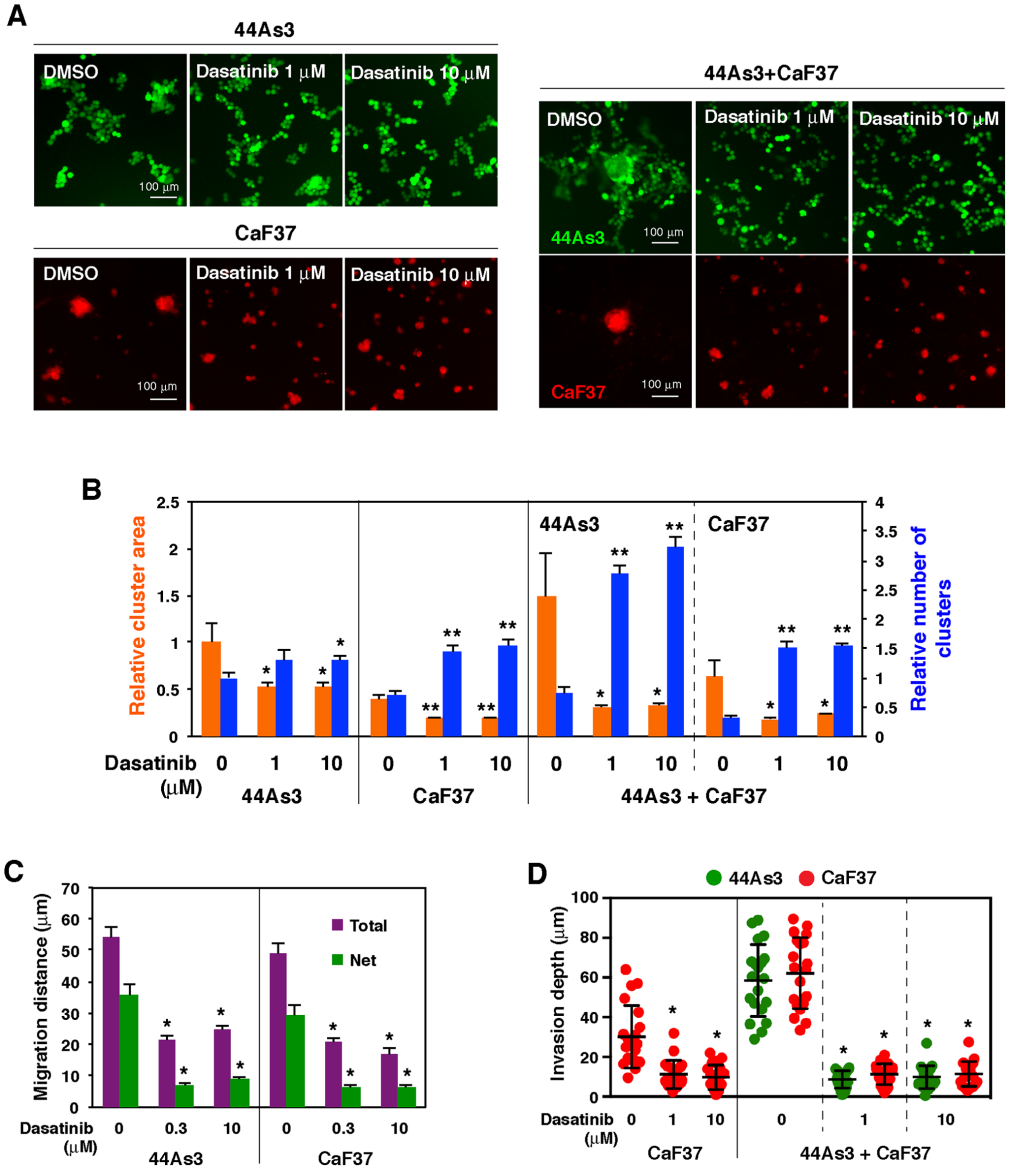
Dasatinib inhibits cell aggregation, migration, and invasion in vitro. A, Fluorescent images of individually cultured and cocultured 44As3 and CaF37 cells on 3D Matrigel in the presence or absence of dasatinib (1 or 10 µM) for 2 days. B, Quantification of the areas and number of cell clusters. Bars show mean ± SEM (*n* = 48–502 for the cluster area, 6 for the number of clusters). *, *p*<0.05; **, *p*<0.001 by Student's *t*-test. C, Total and net migration distances of cells per hour were measured from time-lapse movies. Bars show mean ± SEM (*n* = 40). *, *p*<0.0001 by Student's *t*-test. D, Invasion depth of the invasive foci or cell clusters. Bars show mean ± SD (*n* = 20). *, *p*<0.0001 by Student's *t*-test.

44As3 and CaF37 cells treated with dasatinib remained single cells or small cell clusters on 3D Matrigel and did not efficiently form invasive foci (Fig. 4A and B). Time-lapse imaging demonstrated that cell migration and interaction were markedly inhibited by dasatinib treatment (Video S7). However, in contrast to H1152, dasatinib did not affect MLC phosphorylation (Fig. 4D) nor disruption of gelatin film in 2D coculture condition (Fig. 4E and F). These results indicate that dasatinib inhibits early stages of invasive foci formation, i. e. cell migration and cell-cell interaction, rather than actomyosin-mediated contraction and ECM remodeling. To test this hypothesis, we further characterized the effects of dasatinib. The ability of 44As3 and CaF37 cells to aggregate on 3D Matrigel was analyzed by calculating the areas and number of cell clusters in both individual and coculture conditions (Fig. 5A and B). Dasatinib treatment significantly reduced the areas of cell clusters and concomitantly increased the number of cell clusters in both cell types irrespective of the culture condition. Moreover, cell migration of both 44As3 and CaF37 cells on 3D Matrigel, as determined by calculation of the total and net migration distances from time-lapse movies, was suppressed by dasatinib treatment (Fig. 5C). Invasion depth of cocultured 44As3 and CaF37 cells within invasive foci as well as individually cultured CaF37 cells was also markedly reduced by dasatinib treatment (Fig. 5D). These results suggest that dasatinib impairs cell migration and interaction of SGC and SFs and, therefore, blocks SF-mediated invasion of SGCs.

Dasatinib inhibits Abl kinase activity as well as Src [23]. Nevertheless, we thought the effect of dasatinib is attributed to the inhibition of Src activity, because PP2, another Src inhibitor with different spectrum, also significantly inhibited the formation of invasive foci in our inhibitor library screening (Table S1 and Fig. S4). Consistent with this hypothesis, PP2 effectively blocked the formation of invasive foci as potently as dasatinib, while an Abl inhibitor imatinib exhibited no suppressive effect, rather tended to promote it (Fig. S5A and B). In addition, PP2 and imatinib did not affect disruption of gelatin by 44As3 and CaF37 cells in 2D coculture condition (Fig. S5C and D).

**Figure 6:**
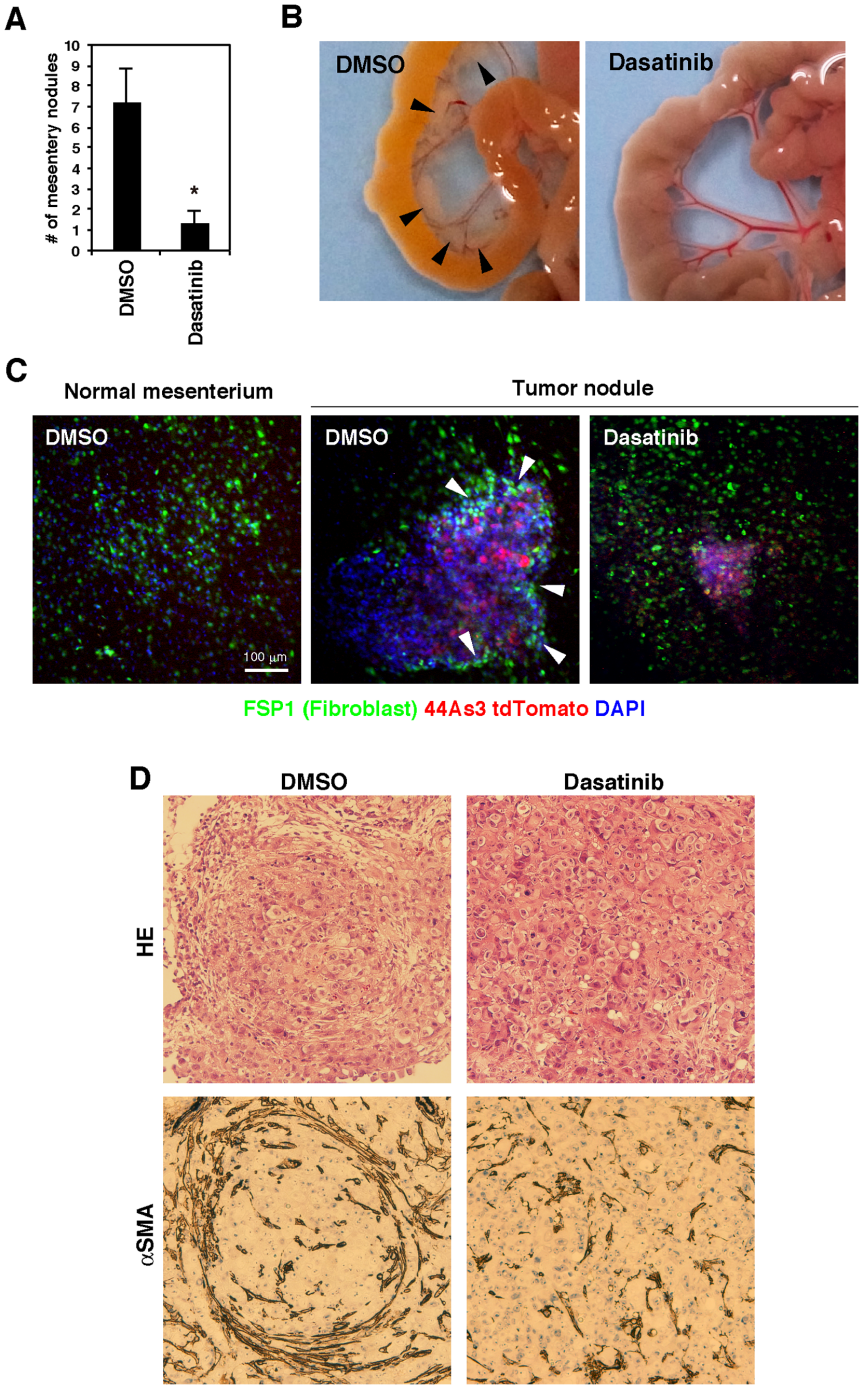
Dasatinib suppresses peritoneal dissemination of SGC cells and their association with stromal fibroblasts in vivo. A, 44As3 cells were intraperitoneally injected into nude mice and DMSO or dasatinib was administered via intraperitoneal injection. The number of mesentery nodules was calculated as described in the Materials and Methods. Bars show mean ± SEM (*n* = 10). *, *p*<0.005 by Mann-Whitney test. B, Representative macroscopic views of metastatic tumor nodules (arrowheads) formed in the mesentery. C, Immunofluorescence analysis of the mouse mesenteries bearing tdTomato-labeled 44As3 tumor nodules. Arrowheads denote the regions where FSP1 positive stromal fibroblasts were accumulated around tumor nodules. D, Mesentery nodules were stained with hematoxylin and eosin and anti-αSMA antibody for histological examination.

Finally, we investigated the effect of dasatinib on peritoneal dissemination of 44As3 cells in nude mice. Dasatinib treatment markedly reduced the number of tumor nodules formed on mesentery (Fig. 6A and B) and also suppressed ascites formation and dissemination to other tissues including omentum, parietal peritoneum, diaphragm, and liver (Table 1). We then performed immunofluorescence analysis of mesentery after injection of tdTomato-labeled 44As3 cells. This analysis revealed that SFs positive for a fibroblast marker FSP1 are accumulated at the boundary regions between tumor nodules and mesentery (Fig. 6C). Such accumulation of SFs was significantly impaired in mesentery treated with dasatinib. Histological analysis also revealed that the mesentery nodules from control mice were strongly associated with SFs positive for αSMA, while those from mice treated with dasatinib contained significantly less SFs (Fig. 6D). These observations suggest that dasatinib treatment inhibits the interaction between SGC cells and SFs in vivo, resulting in a reduced peritoneal dissemination of SGC cells.

**Table 1:** Effect of dasatinib on peritoneal dissemination of 44As3 cells.

Treatment	Ascites	Metastasis				
		Omentum	Mesentery	Parietal Peritoneum	Diaphragm	Liver
DMSO	5/10	10/10	10/10	5/10	5/10	2/10
Dasatinib	0/10	1/10	4/10	0/10	2/10	0/10

Number of mice bearing ascites or tumor at the indicated site per total number of mice.

## Discussion

**Figure 7:**
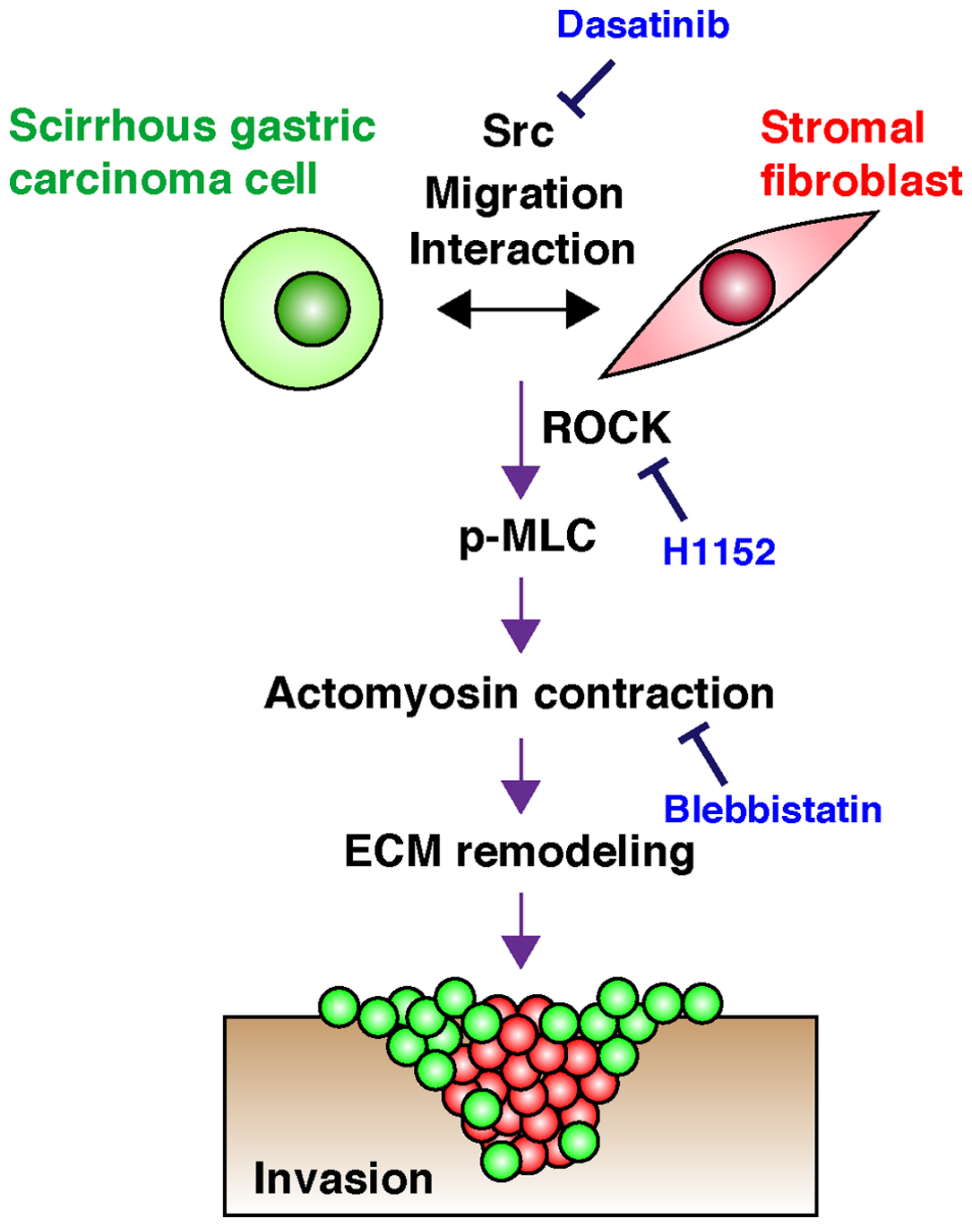
Schematic diagram of the SF-mediated invasion of SGC cells. SGC cells and SFs physically interact with each other through active migration and cell-cell contact, which is dependent on Src activity and therefore blocked by dasatinib treatment. This interaction promotes Rock-dependent phosphorylation of MLC, resulting in actomyosin contraction and mechanical ECM remodeling during invasion of SGC. In peritoneal dissemination, SGC cells may also associate with SFs to invade mesentery and form tumor nodules.

In spite of their highly invasive behavior in vivo, we observed that SGC cells alone had a low invasive ability by themselves on both 3D Matrigel and 2D gelatin matrix. In contrast, SFs had some invasive and ECM remodeling activities that were markedly promoted by coculture with SGC cells. We discovered that SGC cells and SFs form invasive foci in which SFs constitute the core invading structures. In addition, SGC cells were physically attached to SFs in these structures and co-invaded the 3D Matrigel. These results are consistent with a previous report that SFs generate tracks in ECM by mechanical remodeling to initiate and lead invasion of squamous cell carcinoma cells [6]. Therefore, SFs potentially play an essential role in ECM remodeling and mediate invasion of SGC cells (Fig. 7). The increased proliferation of fibroblasts observed within SGC lesions may thus contribute to the rapid infiltration of SGC cells.

Mounting evidence suggests that stromal ECM composition and alterations in its structure foster tumor progression [24]. This process seems to largely depend on secretion, mechanical remodeling, and crosslinking of ECM components by SFs. Considering that the pathological features of SGC include severe fibrosis and resulting stiffening of the gastric wall, these changes in the biophysical properties of the tumor stroma may promote the invasion of SGC cells. Furthermore, SGC sometimes causes massive contractions of the stomach wall in the disease progression. Our results have shown that SGC cells enhance actomyosin-mediated contractility of SFs, resulting in mechanical remodeling and contraction of the ECM. Therefore, SF contraction stimulated by SGC cells may serve multiple roles in the pathogenesis of SGC, such as hardening and contraction of the gastric wall. Since the matrix rigidity is known to support invasiveness of cancer cells [25], enhancement of rigidity of tumors by cooperation between SGC cells and SFs might contribute the invasive properties of SGC.

It has been established that the interaction between cancer cells and SFs is mediated by soluble factors [1]. Indeed, several studies showed that soluble factors, including TGF-β, HGF, and bFGF, mediate stimulation of myofibroblasts phenotypes of SFs by SGC cells [15], [26], as well as enhancement of the growth and invasive phenotypes of SGC cells by SFs [13], [15], [27]–[30]. To the contrary, Semba et al. reported that direct interaction, but not soluble factors, of SGC cells and SFs increases SF proliferation and invasive properties of SGC cells [14]. Consistent with this result, in our experiments conditioned medium from SGC cells could not recapitulate the phenotypes observed in the coculture experiments. These results indicate that a direct association between SGC cells and SFs, in addition to communication via soluble factors, is required for their full biological interactions. Indeed, time-lapse imaging revealed that the 2 cell types communicate with each other by strong physical associations during the formation of invasive foci. Moreover, coculturing SGC cells and SFs did not affect the myofibroblast phenotype as revealed by the unchanged expression of αSMA. Consequently, direct interaction of the 2 cell types leads to the activation of an intracellular signaling pathway that regulates actomyosin contractility, rather than altering the myofibroblast phenotype. We found that Rock is a critical regulator of the formation of invasive foci. Therefore, Rock/MLC signaling seems to play an essential role in mediating ECM remodeling and invasion by SGC and SFs. Next challenge will be to identify molecules directly involved in the physical interaction between SGC cells and SFs. Because VCAM-1 and integrin α4 were previously identified as potential mediators of their direct interaction [14], these molecules may also play a role in the formation of invasive foci and invasive growth and dissemination of SGC.

We observed that Src inhibition by dasatinib significantly blocked the formation of invasive foci in 3D culture by impairing cell migration and resulting physical cell associations (Fig. 7). However, it did not affect mechanical gelatin remodeling by SGC cells and SFs in 2D culture. Because the two cell types were intermingled and in close proximity to each other in 2D culture condition, they may not require Src activity to physically interact. Nevertheless, this idea does not rule out the possibility that Src has distinct functions in SF-mediated invasion. Calvo et al. recently reported that mechanical remodeling and stiffening of ECM by CAFs induces YAP activation via Src-mediated mechanotransduction, which in turn promotes expression of cytoskeletal proteins and contractile phenotypes of CAFs, thus establishing a positive feedback loop [31]. At least in our experimental system, acute effect of dasatinib seems to be the inhibition of the interaction between SGC cells and SFs as described above. However, it is possible that long-term treatment of dasatinib, like in SGC xenograft study in vivo, affects contractile phenotypes of SFs due to decreased transcriptional activity of YAP. Therefore, it will be interesting to explore the role of YAP-dependent matrix stiffening by CAFs in invasive progression and peritoneal dissemination of SGC.

The formation of invasive foci was preferentially observed in SGC cell lines among gastric cancer cell lines, suggesting that this was representative of some of the biological characteristics of SGC cells. Additionally, we demonstrated that invasion of SGC cells occurred only when they formed invasive foci with SFs. Therefore, analysis of the formation of invasive foci may be useful for the evaluation and quantification of SF-mediated invasion and the ECM remodeling activity of SGC cells. Indeed, we utilized this assay system to screen inhibitor library and identified dasatinib as a potent inhibitor of the interaction between SGC cells and SFs. Moreover, we demonstrated for the first time that dasatinib blocks accumulation of SFs around and within mesentery tumor nodules and effectively reduces peritoneal dissemination of SGC cells in mice. Peritoneal dissemination of SGC is associated with strong fibrosis at the metastasis sites and is a critical and poor prognostic factor [10]. Therefore, SFs may also support invasion of SGC cells into mesentery stroma and resulting formation of metastasis. Taken together, this experimental system may enhance the study of the biological interactions between SGC cells and SFs, allowing the identification of regulating compounds, and thus facilitate the development of new therapeutics for SGC.

## Supporting Information

Figure S1 **Characterization of the formation of invasive foci.** A, Invasion depth of invasive foci formed by 44As3 and CaF37 cells on 3D Matrigel. Bars show mean ± SD (n = 20). *, *p*<0.0001 by Student's t-test. B, CaF37 cells were cultured either with 44As3 cells or conditioned medium (CM) of 44As3 cells on 3D Matrigel for 2 days. 44As3 cells were also cultured with conditioned medium of CaF37 cells. C, A representative scanned image of invasive foci formed by 44As3 and CaF37 cells on 3D Matrigel. D, The number of invasive foci was quantified and shown as the relative values. Bars show mean ± SEM (*n* = 4). *, *p*<0.0005; **, *p*<0.000001 by Student's *t*-test.
(TIF)

Figure S2 **Cocultured 44As3 and CaF38 cells form invasive foci on 3D Matrigel and remodel gelatin matrix.** A, Invasive foci formed by 44As3 and CaF38 cells. B, Relative number of invasive foci. Bars show mean ± SEM (*n* = 4). *, *p*<0.01 by Student's *t*-test. C, Gelatin remodeling activity of 44As3 and CaF38 cells. D, The areas of gelatin detachment were quantified and shown as relative values. Bars show mean ± SEM (*n* = 5). *, *p*<0.001 by Student's *t*-test.
(TIF)

Figure S3 **Formation of invasive foci and remodeling of ECM by 44As3 and CaF37 cells were not blocked by GM6001.** A, Gelatin remodeling activity of 44As3 and CaF37 cells in the absence or presence of GM6001 (10 µM). B, The areas of gelatin disruption. Bars show mean ± SEM (*n* = 4). C, MDA-MB-231 cells were cultured on fluorescent gelatin-coated cover slips in the absence or presence of GM6001 (10 µM) for 7 h. D, Formation of invasive foci by 44As3 and CaF37 cells in the absence or presence of GM6001 (10 µM). E, The relative number of invasive foci. Bars show mean ± SEM (*n*s = 4).
(TIF)

Figure S4 **Representative images for inhibitor library screening.** CellTracker-labeled 44As3 and CaF37 cells were cultured on 3D Matrigel in the absence or presence of indicated inhibitors (10 µM) for 2 days and observed by confocal microscopy.
(TIF)

Figure S5 **Effect of PP2 and imatinib on the formation of invasive foci and gelatin remodeling by cocultured 44As3 and CaF37 cells.** A, The effect of PP2 (10 µM) and imatinib (10 µM) on invasive foci formation by 44As3 and CaF37 cells. B, The relative number of invasive foci. Bars show mean ± SEM (*n* = 5 for PP2 and 3 for imatinib). *, *p*<0.00005 by Student's *t*-test. C, The effect of PP2 (10 µM) and imatinib (10 µM) on gelatin remodeling activity of 44As3 and CaF37 cells. D, The areas of gelatin disruption. Bars show mean ± SEM (*n* = 3).
(TIF)

Video S1 **Formation of invasive foci by 44As3 and CaF37 cells.** 44As3 and CaF37 cells were labeled with CellTracker Green and Red, respectively, and plated onto 3D Matrigel. The cells were imaged every 5 min by time-lapse fluorescence microscopy for 16 h. Play rate, 15 frames/sec. Still images are shown in Figure 2A.
(MOV)

Video S2 **Remodeling of Matrigel by 44As3 cells.** 44As3 cells were cultured on 3D Matrigel containing fluorescent microbeads. The cells and microbeads were imaged every 5 min by time-lapse fluorescence microscopy for 8 h 45 min. Play rate, 15 frames/sec.
(MOV)

Video S3 **Remodeling of Matrigel by CaF37 cells.** CellTracker Green-labeled CaF37 cells were cultured and imaged as in Video S2.
(MOV)

Video S4 **Remodeling of Matrigel by 44As3 and CaF37 cells.** 44As3 cells and CellTracker Green-labeled CaF37 cells were cocultured and imaged as in Video S2.
(MOV)

Video S5 **Formation of invasive foci in the presence of DMSO (control).** CellTracker Green-labeled 44As3 cells and CellTracker Orange-labeled CaF37 cells were cocultured on 3D Matrigel in the presence of DMSO. The cells were imaged every 5 min for 14 h 45 min. Play rate, 15 frames/sec.
(MOV)

Video S6 **H1152 impairs formation of invasive foci.** Cells were cultured and imaged as in Video S5 in the presence of H1152 (10 µM).
(MOV)

Video S7 **Dasatinib impairs formation of invasive foci.** Cells were cultured and imaged as in Video S5 in the presence of dasatinib (10 µM).
(MOV)

Table S1 **List of inhibitors screened and their effects on the formation of invasive foci.** The relative number of invasive foci and cytotoxicity against 44As3 and CaF37 cells are shown for each compound in the screen at 10 µM. Dasatinib and H1152 are highlighted in red.
(DOCX)
