# Supplementary figures and images for: Stromal Fibroblasts Mediate Extracellular Matrix Remodeling and Invasion of Scirrhous Gastric Carcinoma Cells
Source: PLoS One. 2014 Jan 10;9(1):e85485. doi: 10.1371/journal.pone.0085485 (PMC3888433; doi:10.1371/journal.pone.0085485)

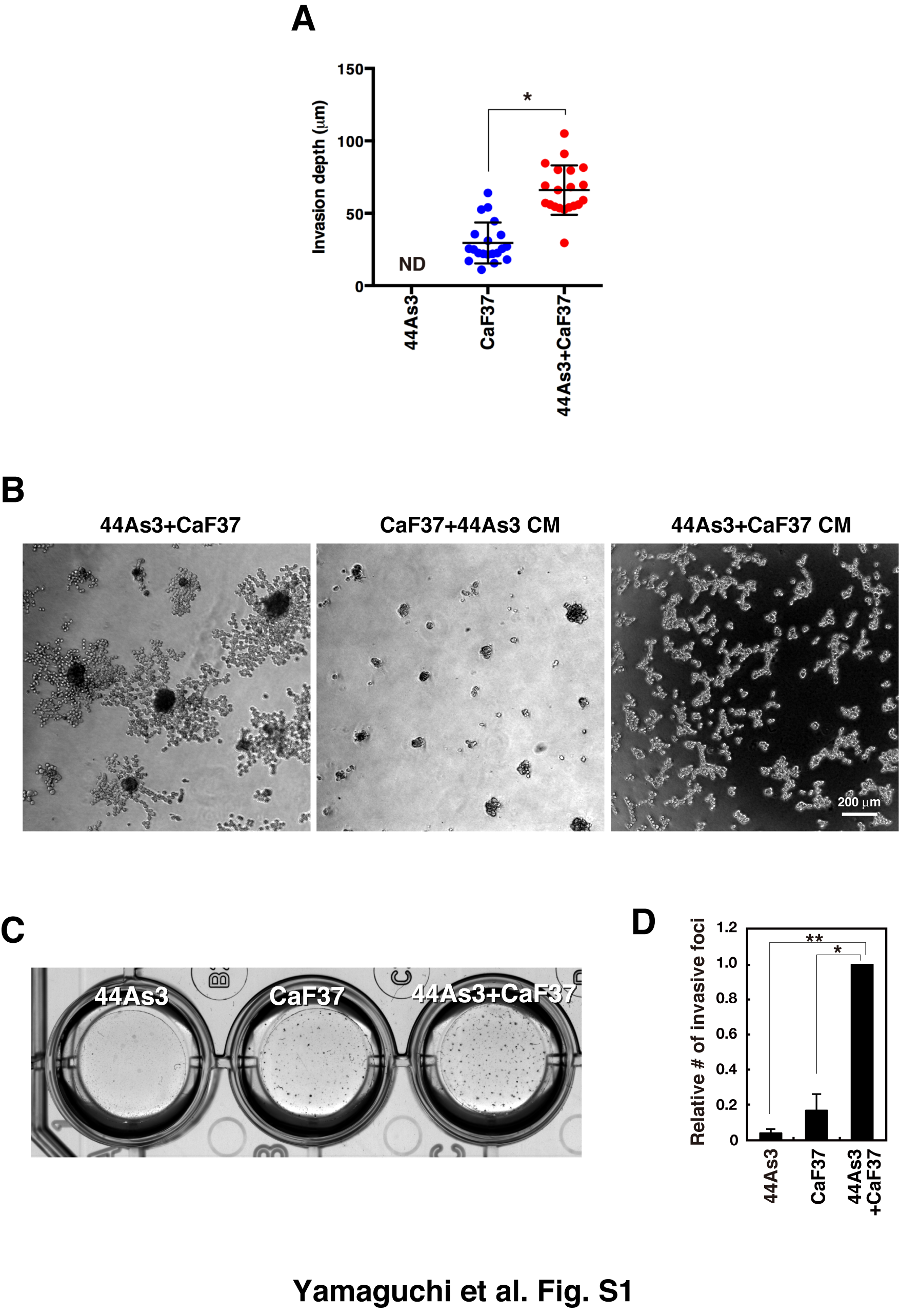

Supplement: Figure S1 — Characterization of the formation of invasive foci. A, Invasion depth of invasive foci formed by 44As3 and CaF37 cells on 3D Matrigel. Bars show mean ± SD (n = 20). *, p<0.0001 by Student's t-test. B, CaF37 cells were cultured either with 44As3 cells or conditioned medium (CM) of 44As3 cells on 3D Matrigel for 2 days. 44As3 cells were also cultured with conditioned medium of CaF37 cells. C, A representative scanned image of invasive foci formed by 44As3 and CaF37 cells on 3D Matrigel. D, The number of invasive foci was quantified and shown as the relative values. Bars show mean ± SEM (n = 4). *, p<0.0005; **, p<0.000001 by Student's t-test. (TIF) [file pone.0085485.s001.tif]

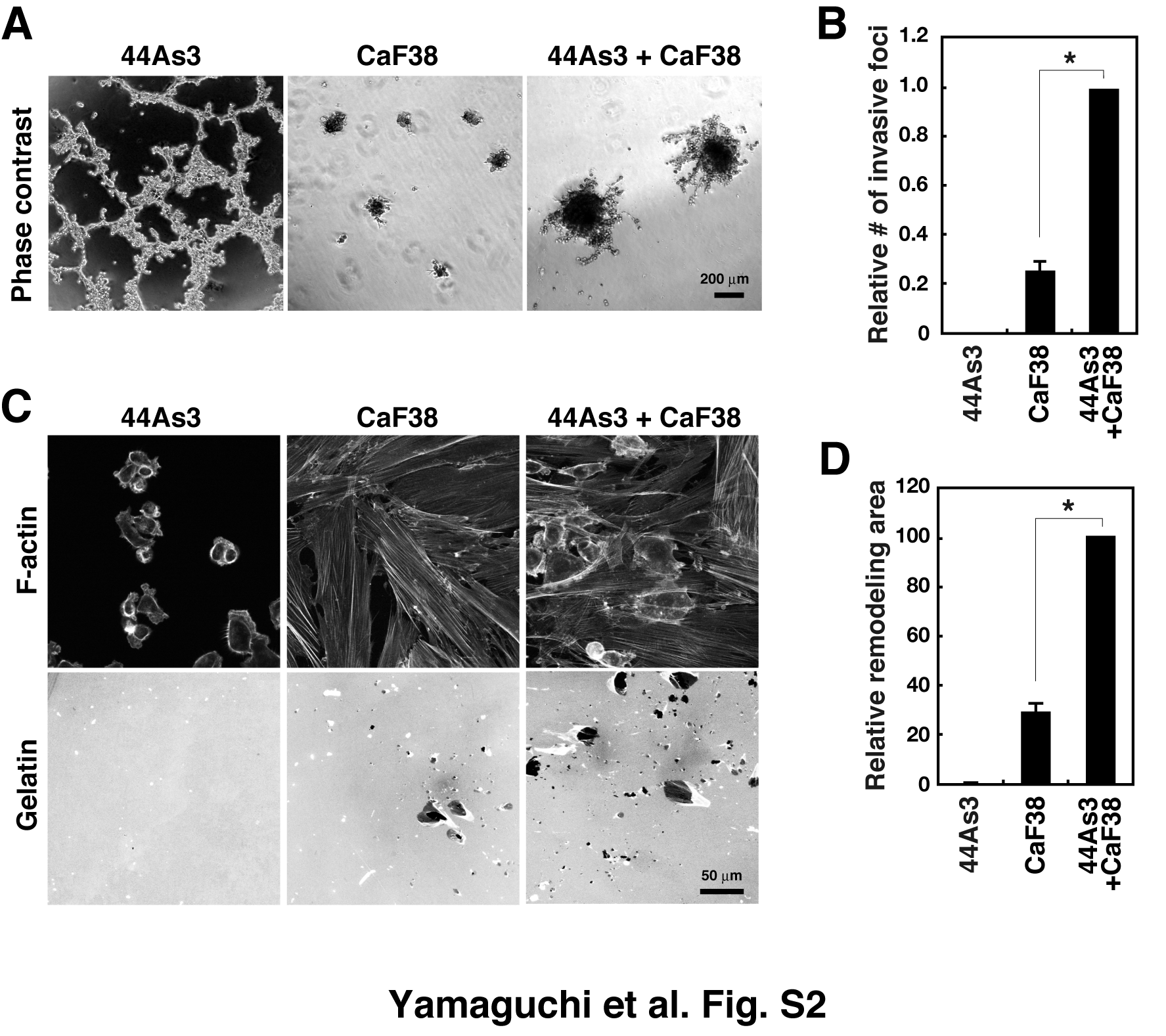

Supplement: Figure S2 — Cocultured 44As3 and CaF38 cells form invasive foci on 3D Matrigel and remodel gelatin matrix. A, Invasive foci formed by 44As3 and CaF38 cells. B, Relative number of invasive foci. Bars show mean ± SEM (n = 4). *, p<0.01 by Student's t-test. C, Gelatin remodeling activity of 44As3 and CaF38 cells. D, The areas of gelatin detachment were quantified and shown as relative values. Bars show mean ± SEM (n = 5). *, p<0.001 by Student's t-test. (TIF) [file pone.0085485.s002.tif]

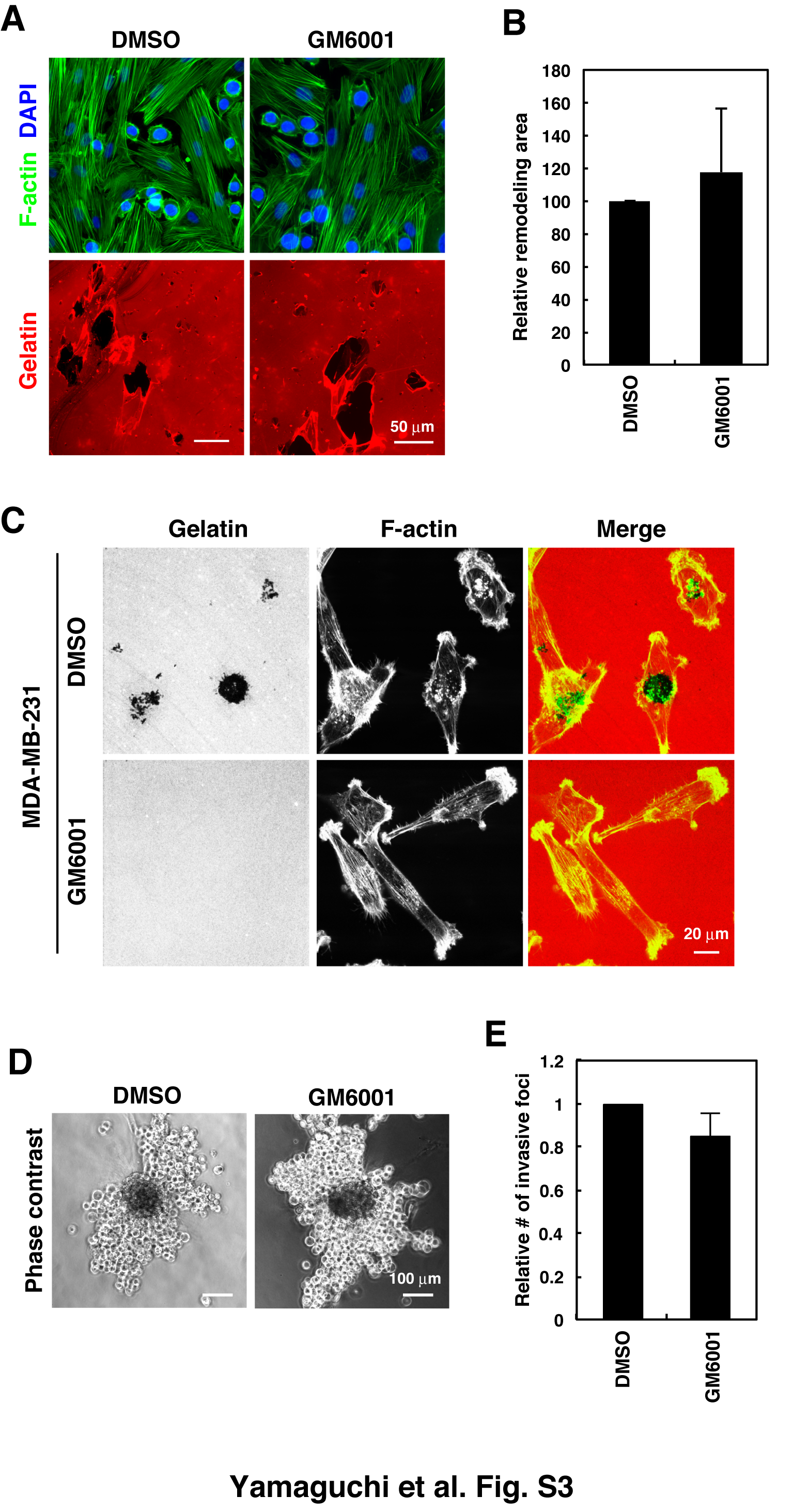

Supplement: Figure S3 — Formation of invasive foci and remodeling of ECM by 44As3 and CaF37 cells were not blocked by GM6001. A, Gelatin remodeling activity of 44As3 and CaF37 cells in the absence or presence of GM6001 (10 µM). B, The areas of gelatin disruption. Bars show mean ± SEM (n = 4). C, MDA-MB-231 cells were cultured on fluorescent gelatin-coated cover slips in the absence or presence of GM6001 (10 µM) for 7 h. D, Formation of invasive foci by 44As3 and CaF37 cells in the absence or presence of GM6001 (10 µM). E, The relative number of invasive foci. Bars show mean ± SEM (ns = 4). (TIF) [file pone.0085485.s003.tif]

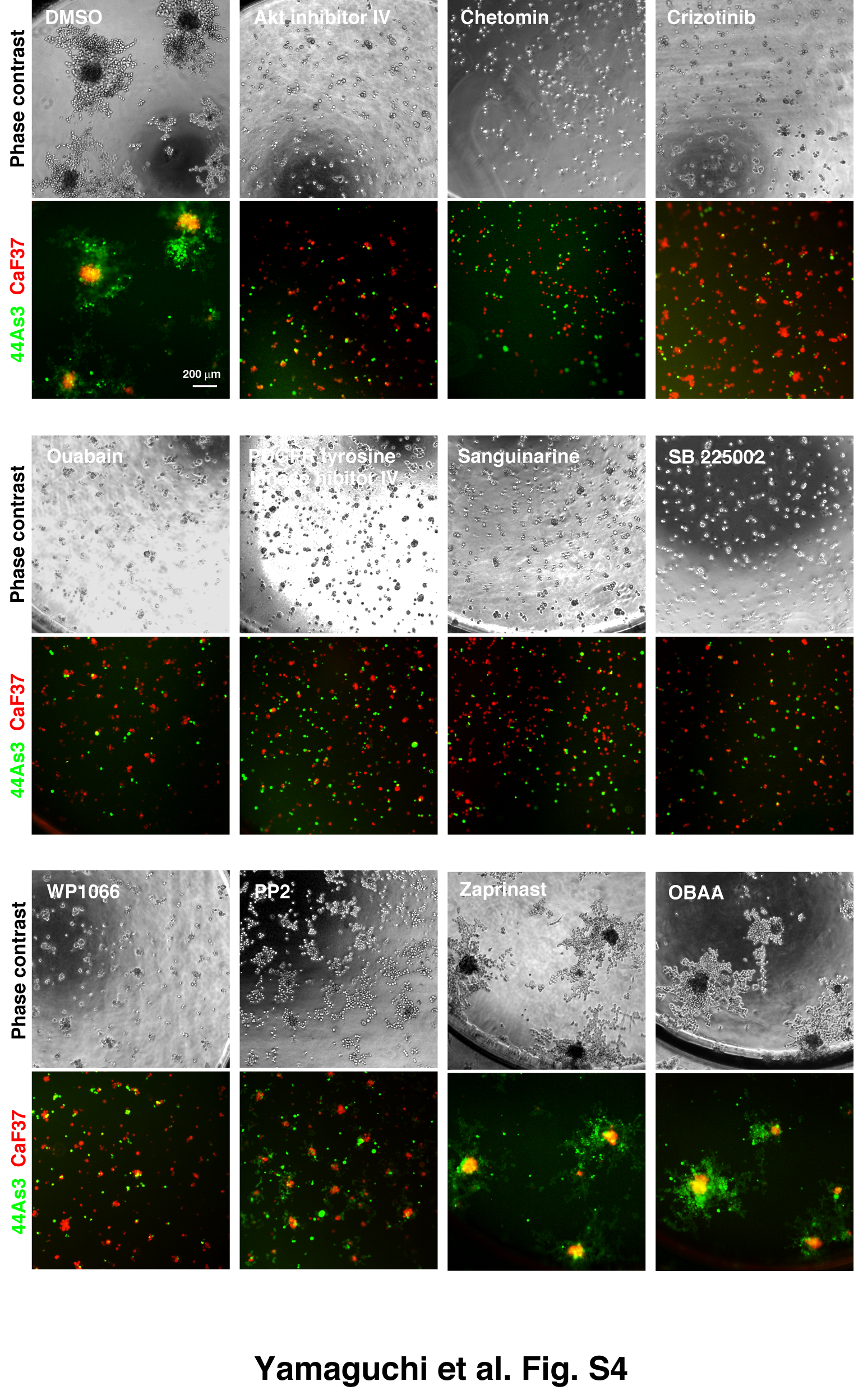

Supplement: Figure S4 — Representative images for inhibitor library screening. CellTracker-labeled 44As3 and CaF37 cells were cultured on 3D Matrigel in the absence or presence of indicated inhibitors (10 µM) for 2 days and observed by confocal microscopy. (TIF) [file pone.0085485.s004.tif]

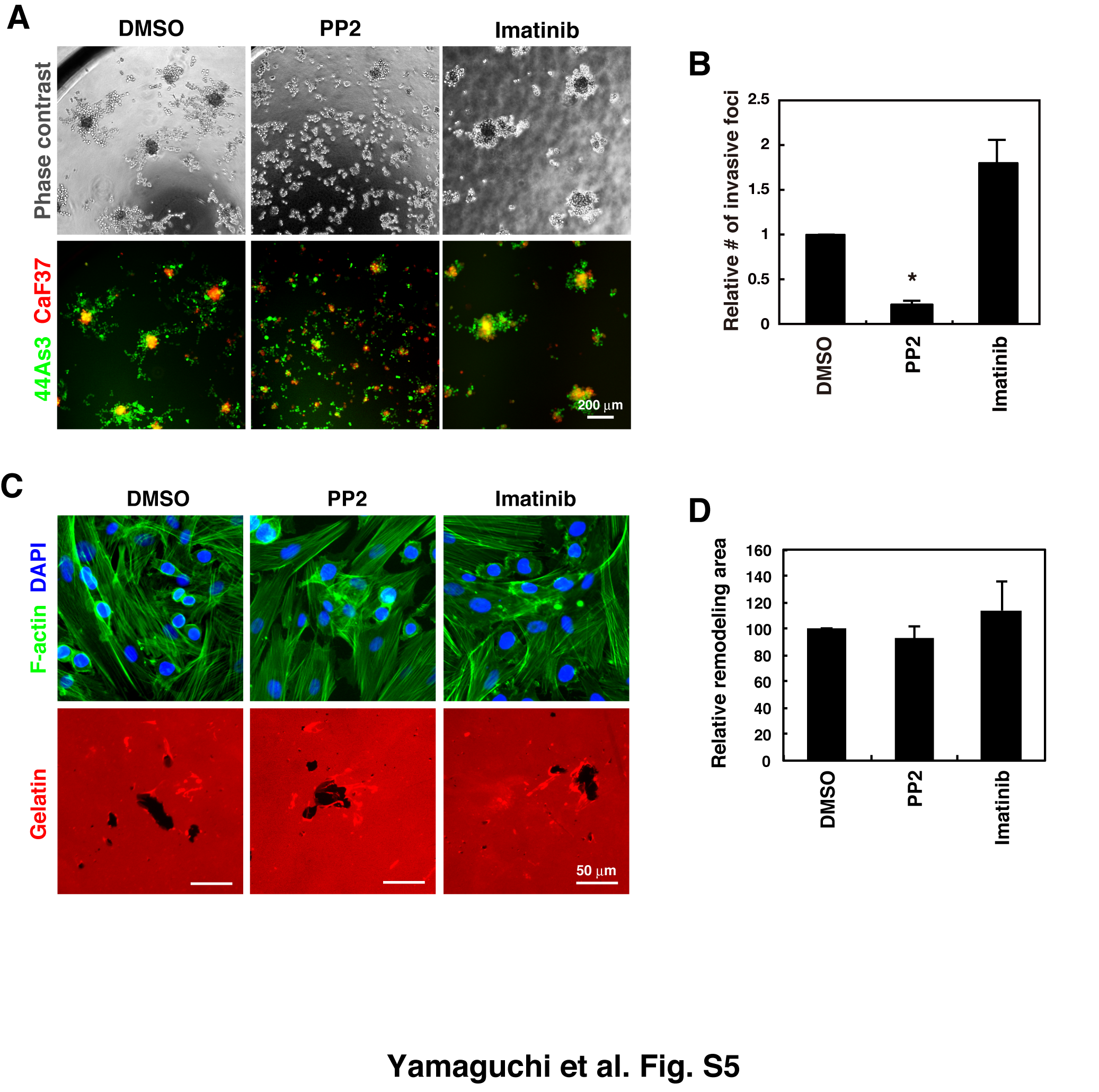

Supplement: Figure S5 — Effect of PP2 and imatinib on the formation of invasive foci and gelatin remodeling by cocultured 44As3 and CaF37 cells. A, The effect of PP2 (10 µM) and imatinib (10 µM) on invasive foci formation by 44As3 and CaF37 cells. B, The relative number of invasive foci. Bars show mean ± SEM (n = 5 for PP2 and 3 for imatinib). *, p<0.00005 by Student's t-test. C, The effect of PP2 (10 µM) and imatinib (10 µM) on gelatin remodeling activity of 44As3 and CaF37 cells. D, The areas of gelatin disruption. Bars show mean ± SEM (n = 3). (TIF) [file pone.0085485.s005.tif]
