# Supplementary material for: Stromal Fibroblasts Mediate Extracellular Matrix Remodeling and Invasion of Scirrhous Gastric Carcinoma Cells
Source: PLoS One. 2014 Jan 10;9(1):e85485. doi: 10.1371/journal.pone.0085485 (PMC3888433; doi:10.1371/journal.pone.0085485)
Supplement: Table S1 — List of inhibitors screened and their effects on the formation of invasive foci. The relative number of invasive foci and cytotoxicity against 44As3 and CaF37 cells are shown for each compound in the screen at 10 µM. Dasatinib and H1152 are highlighted in red. (DOCX) [file pone.0085485.s013.docx]

Table S1. List of inhibitors screened and their effects on the formation of invasive foci

The relative number of invasive foci and cytotoxicity against 44As3 and CaF37 cells are shown for each compound in the screen at 10 μM. Dasatinib and H1152 are highlighted in red.

| Relative number of invasive foci | Compound | 44As3 Cytotoxicity | CaF37 Cytotoxicity |
| --- | --- | --- | --- |
| 0.00 | Akt Inhibitor IV | 0.82 | 1.22 |
| 0.00 | Chetomin | 0.72 | 1.05 |
| 0.00 | crizotinib | 0.21 | 1.12 |
| 0.00 | dasatinib | 0.04 | 0.01 |
| 0.00 | Ouabain | 0.60 | 0.99 |
| 0.00 | PDGF receptor tyrosine kinase inhibitor IV | 0.22 | 0.05 |
| 0.00 | Sanguinarine | 0.91 | 1.12 |
| 0.00 | SB 225002 | 0.01 | 0.07 |
| 0.00 | WP1066 | 0.62 | 1.01 |
| 0.08 | Rotenone | 0.14 | 0.05 |
| 0.10 | Aurora kinase/cdk inhibitor | 0.00 | 0.54 |
| 0.10 | Cdk1/2 inhibitor III | 0.06 | 1.06 |
| 0.10 | Cdk2/9 inhibitor | 0.10 | 0.30 |
| 0.10 | IKK-2 inhibitor VI | 0.00 | 0.00 |
| 0.10 | SU11652 | 0.22 | 1.14 |
| 0.11 | Cantharidin | 0.64 | 1.18 |
| 0.11 | Cucurbitacin I | 0.16 | 0.99 |
| 0.11 | Cytochalasin D | 0.00 | 0.10 |
| 0.11 | Nocodazole | 0.37 | 0.07 |
| 0.13 | bortezomib | 0.07 | 0.65 |
| 0.16 | Nordihydroguaiaretic acid (NDGA) | 0.14 | 0.00 |
| 0.19 | Akt Inhibitor XI | 0.00 | 0.00 |
| 0.19 | H-1152 | 0.06 | 0.00 |
| 0.19 | PP2 | 0.03 | 0.00 |
| 0.22 | Staurosporine | 0.00 | 1.30 |
| 0.25 | Nigericin | 0.34 | 0.11 |
| 0.33 | Ionomycin | 0.73 | 0.13 |
| 0.33 | Actinomycin D | 0.27 | 0.89 |
| 0.33 | Vinblastine sulfate | 0.44 | 0.08 |
| 0.38 | pazopanib | 0.00 | 0.06 |
| 0.38 | Torkinib | 0.14 | 0.01 |
| 0.38 | D4476 | 0.00 | 0.00 |
| 0.38 | Diacylglycerol kinase inhibitor II | 0.17 | 0.00 |
| 0.38 | SU4984 | 0.01 | 0.00 |
| 0.38 | TrkA inhibitor | 0.00 | 0.00 |
| 0.39 | Wortmannin | 0.03 | 0.03 |
| 0.41 | A23187 | 0.75 | 0.70 |
| 0.41 | LY 83583 | 0.29 | 0.39 |
| 0.41 | AG1296 | 0.00 | 0.00 |
| 0.43 | LY294002 | 0.12 | 0.00 |
| 0.44 | Cycloheximide | 0.08 | 0.00 |
| 0.44 | Paclitaxel | 0.11 | 0.00 |
| 0.45 | Y-27632 | 0.00 | 0.00 |
| 0.48 | BPIQ-Ⅱ | 0.00 | 0.00 |
| 0.48 | MEK inhibitor I | 0.05 | 0.00 |
| 0.48 | TX-1918 | 0.00 | 0.00 |
| 0.49 | ETYA | 0.15 | 0.03 |
| 0.49 | SB 328437 | 0.00 | 0.05 |
| 0.49 | Radicicol | 0.13 | 0.00 |
| 0.54 | SP600125 | 0.21 | 0.00 |
| 0.56 | HA1077 | 0.17 | 0.00 |
| 0.56 | Z-VAD-FMK | 0.00 | 0.00 |
| 0.57 | Dequalinium | 0.63 | 0.12 |
| 0.57 | MK 886 | 0.00 | 0.05 |
| 0.57 | NSC95397 | 0.07 | 0.02 |
| 0.57 | Swainsonine | 0.01 | 0.02 |
| 0.57 | Troglitazone | 0.00 | 0.00 |
| 0.57 | H-89 | 0.21 | 0.00 |
| 0.58 | Chk2 inhibitor II | 0.14 | 0.00 |
| 0.58 | isogranulatimide | 0.04 | 0.00 |
| 0.58 | JNK inhibitor VIII | 0.03 | 0.00 |
| 0.58 | PDGF receptor tyrosine kinase inhibitor V | 0.06 | 0.00 |
| 0.58 | SU5402 | 0.02 | 0.00 |
| 0.58 | VEGFR receptor tyrosine kinase inhibitor II | 0.00 | 0.00 |
| 0.63 | imatinib mesylate | 0.68 | 1.23 |
| 0.63 | Leptomycin B* | 0.04 | 0.17 |
| 0.63 | nilotinib | 0.00 | 0.00 |
| 0.63 | PIM1/2 Kinase Inhibitor V | 0.00 | 0.03 |
| 0.65 | a-Amanitin | 0.51 | 0.37 |
| 0.65 | Dioctanoylglycol | 0.00 | 0.02 |
| 0.65 | MDM2 inhibitor | 0.04 | 0.03 |
| 0.65 | Ro 5-4864 | 0.06 | 0.05 |
| 0.65 | Tunicamycin | 0.21 | 0.10 |
| 0.67 | FTI-276 | 0.00 | 0.00 |
| 0.67 | Mitomycin C | 0.10 | 0.00 |
| 0.67 | PD 98059 | 0.12 | 0.00 |
| 0.67 | Valeryl salicylate | 0.00 | 0.00 |
| 0.67 | BMS-345541 | 0.01 | 0.07 |
| 0.67 | Chk2 inhibitor | 0.13 | 0.00 |
| 0.67 | KN-62 | 0.00 | 0.00 |
| 0.67 | PD98059 | 0.00 | 0.00 |
| 0.67 | SB202190 | 0.00 | 0.00 |
| 0.69 | Olomoucine | 0.12 | 0.00 |
| 0.73 | BH3I-1 | 0.00 | 0.02 |
| 0.73 | Clofibrate | 0.02 | 0.05 |
| 0.73 | Fumonisin B1 | 0.00 | 0.00 |
| 0.73 | N-phenylanthranilic acid | 0.09 | 0.06 |
| 0.75 | everolimus | 0.37 | 0.00 |
| 0.75 | IWR-1-endo | 0.00 | 0.00 |
| 0.75 | Thapsigargin | 0.78 | 1.11 |
| 0.77 | 4-cyano-3-methylisoquinoline | 0.04 | 0.00 |
| 0.77 | AGL 2263 | 0.01 | 0.00 |
| 0.77 | Aurora kinase inhibitor III | 0.00 | 0.00 |
| 0.77 | IC60211 | 0.01 | 0.00 |
| 0.77 | Indirubin-3'-monoxime | 0.12 | 0.00 |
| 0.77 | JAK Inhibitor I | 0.03 | 0.00 |
| 0.77 | NU6102 | 0.00 | 0.00 |
| 0.78 | 17-AAG | 0.00 | 0.00 |
| 0.78 | Cyclosporin A | 0.33 | 0.00 |
| 0.78 | Doxorubicin, HCl | 0.28 | 0.88 |
| 0.78 | MG-132 | 0.29 | 0.57 |
| 0.78 | Sulindac sulfide | 0.03 | 0.00 |
| 0.81 | AZT | 0.00 | 0.00 |
| 0.81 | BADGE | 0.00 | 0.03 |
| 0.81 | Decylubiquinone | 0.19 | 0.02 |
| 0.81 | DIDS | 0.00 | 0.03 |
| 0.81 | Nalidixic acid | 0.00 | 0.00 |
| 0.81 | Nifedipine | 0.00 | 0.00 |
| 0.82 | AG1478 | 0.00 | 0.00 |
| 0.86 | AG490 | 0.20 | 0.00 |
| 0.86 | Lavendustin C | 0.00 | 0.00 |
| 0.86 | Tpl2 kinase inhibitor | 0.00 | 0.00 |
| 0.88 | Olaparib | 0.11 | 0.00 |
| 0.89 | AKT inhibitor | 0.07 | 0.00 |
| 0.89 | Aminoguanidine, HCl | 0.01 | 0.00 |
| 0.89 | Benzamide | 0.08 | 0.00 |
| 0.89 | Bleomycin sulfate | 0.03 | 0.00 |
| 0.89 | Methotrexate | 0.00 | 0.00 |
| 0.89 | NS-398 | 0.00 | 0.00 |
| 0.89 | Rapamycin | 0.31 | 0.00 |
| 0.90 | HA 14-1 | 0.00 | 0.02 |
| 0.90 | Lonidamine | 0.08 | 0.07 |
| 0.90 | MST-312 | 0.47 | 0.02 |
| 0.90 | Oligomycin | 0.73 | 1.06 |
| 0.90 | Z-GLF-CMK | 0.23 | 0.03 |
| 0.91 | LFM-A13 | 0.00 | 0.00 |
| 0.93 | U-0126 | 0.00 | 0.00 |
| 0.96 | Aurora kinase inhibitor II | 0.00 | 0.00 |
| 0.96 | Flt-3 Inhibitor | 0.06 | 0.00 |
| 0.96 | JAK3 Inhibitor VI | 0.10 | 0.90 |
| 0.96 | SB239063 | 0.00 | 0.00 |
| 0.98 | Diazoxide | 0.00 | 0.01 |
| 0.98 | Formestane | 0.00 | 0.00 |
| 0.98 | Fumitremorgin C | 0.01 | 0.05 |
| 0.98 | Mifepristone | 0.01 | 0.00 |
| 0.98 | N1,N12-Diethylspermine (BESpm) | 0.00 | 0.00 |
| 0.98 | PRIMA-1 | 0.00 | 0.00 |
| 0.98 | RHC80267 | 0.00 | 0.00 |
| 0.98 | Xanthohumol | 0.69 | 0.02 |
| 0.98 | Damnacanthal | 0.05 | 0.00 |
| 0.99 | Terreic acid | 0.14 | 0.00 |
| 1.00 | 1400W, HCl | 0.08 | 0.00 |
| 1.00 | Aclarubicin | 0.16 | 0.01 |
| 1.00 | Aphidicolin | 0.10 | 0.00 |
| 1.00 | FH535 | 0.00 | 0.02 |
| 1.00 | GSK-3 inhibitor II | 0.18 | 0.00 |
| 1.00 | H-7 | 0.06 | 0.00 |
| 1.00 | IBMX | 0.00 | 0.00 |
| 1.00 | Monastrol | 0.01 | 0.00 |
| 1.00 | OBAA | 0.10 | 0.00 |
| 1.00 | temozolomide | 0.03 | 0.00 |
| 1.00 | temsirolimus | 0.43 | 1.27 |
| 1.00 | Zaprinast | 0.11 | 0.00 |
| 1.05 | KT5823 | 0.00 | 0.00 |
| 1.05 | Alsterpaullone, 2-cyanoethyl | 0.12 | 0.22 |
| 1.05 | IRAK-1/4 inhibitor | 0.00 | 0.00 |
| 1.05 | SU6656 | 0.06 | 0.00 |
| 1.05 | TGF-b RI kinase inhibitor II | 0.07 | 0.00 |
| 1.06 | C75 | 0.13 | 0.05 |
| 1.06 | HR22C16 | 0.27 | 0.02 |
| 1.06 | ODQ | 0.00 | 0.00 |
| 1.06 | Phenelzine | 0.02 | 0.01 |
| 1.06 | RS 102895 | 0.03 | 0.06 |
| 1.06 | Valinomycin | 0.27 | 0.07 |
| 1.11 | Camptothecin | 0.28 | 0.01 |
| 1.11 | cPLA2inhibitor | 0.09 | 0.00 |
| 1.11 | D609 | 0.02 | 0.00 |
| 1.11 | Dephostatin | 0.08 | 0.00 |
| 1.11 | N-Acetyl-L-cysteine | 0.07 | 0.00 |
| 1.13 | CCG-1423 | 0.01 | 0.02 |
| 1.13 | PIM1 Inhibitor II | 0.00 | 0.13 |
| 1.13 | AG1024 | 0.00 | 0.00 |
| 1.13 | SB431542 | 0.03 | 0.00 |
| 1.14 | Aminoglutethimide | 0.00 | 0.02 |
| 1.14 | Benzylguanine | 0.00 | 0.00 |
| 1.14 | CA-074 | 0.00 | 0.04 |
| 1.14 | DFMO | 0.00 | 0.00 |
| 1.14 | Monensin | 0.60 | 0.18 |
| 1.14 | TOFA | 0.03 | 0.00 |
| 1.14 | Rp-8-CPT-cGMPS | 0.10 | 0.00 |
| 1.15 | PP1 analog | 0.00 | 0.00 |
| 1.15 | 1-Azakenpaullone | 0.41 | 0.00 |
| 1.15 | ABT-702 | 0.00 | 0.00 |
| 1.15 | ATM/ATR kinase inhibitor | 0.00 | 0.00 |
| 1.15 | DMAT | 0.16 | 0.00 |
| 1.15 | ERK inhibitor II | 0.00 | 0.00 |
| 1.15 | GSK-3 inhibitor IX | 0.19 | 0.00 |
| 1.15 | PKR inhibitor | 0.13 | 0.39 |
| 1.17 | AG957 | 0.09 | 0.00 |
| 1.22 | Debromohymenialdisine (DBH) | 0.06 | 0.00 |
| 1.22 | Dimethyloxalylglycine | 0.05 | 0.00 |
| 1.22 | Dexamethasone | 0.11 | 0.00 |
| 1.22 | FK-506 | 0.15 | 0.00 |
| 1.22 | Fumagillin | 0.09 | 0.00 |
| 1.22 | Genistein | 0.01 | 0.00 |
| 1.22 | GM 6001 | 0.16 | 0.00 |
| 1.22 | Manumycin A | 0.06 | 0.00 |
| 1.22 | NU1025 | 0.14 | 0.00 |
| 1.22 | Scriptaid | 0.15 | 0.34 |
| 1.25 | RAF1 kinase inhibitor I | 0.00 | 0.00 |
| 1.25 | Syk inhibitor | 0.04 | 0.00 |
| 1.25 | VEGF recptor 2 kinase inhibitor I | 0.00 | 0.00 |
| 1.25 | Deoxynojirimycin | 0.00 | 0.00 |
| 1.25 | erlotinib | 0.00 | 0.00 |
| 1.30 | TBB | 0.00 | 0.00 |
| 1.30 | Amastatin | 0.00 | 0.00 |
| 1.30 | Baicalein | 0.25 | 0.00 |
| 1.30 | Deprenyl | 0.02 | 0.00 |
| 1.30 | Finasteride | 0.00 | 0.01 |
| 1.30 | Glibenclamide | 0.00 | 0.00 |
| 1.30 | Lidocaine | 0.00 | 0.04 |
| 1.30 | PGP-4008 | 0.08 | 0.12 |
| 1.30 | t-Butylhydroquinone (BHQ) | 0.02 | 0.06 |
| 1.30 | Verapamil | 0.01 | 0.00 |
| 1.32 | Kenpaullone | 0.22 | 0.00 |
| 1.33 | Etoposide (VP-16) | 0.02 | 0.00 |
| 1.33 | Flutamide | 0.00 | 0.00 |
| 1.33 | GGTI-286 | 0.25 | 0.00 |
| 1.33 | Lactacystin | 0.20 | 0.02 |
| 1.33 | Trichostatin A | 0.16 | 0.46 |
| 1.34 | TG003 | 0.07 | 0.00 |
| 1.35 | purvalanol A | 0.21 | 0.10 |
| 1.38 | 5,15-DPP | 0.01 | 0.00 |
| 1.38 | anisomycin | 0.15 | 0.26 |
| 1.38 | IWP-2 | 0.00 | 0.00 |
| 1.38 | sorafenib | 0.06 | 0.00 |
| 1.38 | tamibarotene | 0.07 | 0.00 |
| 1.38 | Anacardic acid | 0.00 | 0.00 |
| 1.38 | E-64d | 0.05 | 0.00 |
| 1.44 | 5-FU | 0.00 | 0.00 |
| 1.44 | AMT, HCl | 0.05 | 0.00 |
| 1.44 | SB 203580 | 0.16 | 0.00 |
| 1.44 | Theophylline | 0.11 | 0.00 |
| 1.47 | Actinonin | 0.03 | 0.01 |
| 1.47 | Amiloride | 0.02 | 0.03 |
| 1.47 | Cerulenin | 0.09 | 0.04 |
| 1.47 | Diltiazem | 0.00 | 0.06 |
| 1.47 | Pepstatin A | 0.00 | 0.02 |
| 1.47 | Pifithrin-a (cyclic) | 0.00 | 0.00 |
| 1.47 | SC-ααδ9 | 0.00 | 0.03 |
| 1.49 | SU1498 | 0.01 | 0.00 |
| 1.50 | thalidomide | 0.08 | 0.00 |
| 1.50 | tretinoin | 0.11 | 0.00 |
| 1.53 | Cdk4 inhibitor | 0.00 | 0.00 |
| 1.53 | cFMS Receptor Tyrosine Kinase Inhibitor | 0.10 | 0.00 |
| 1.53 | ZM 336372 | 0.00 | 0.00 |
| 1.55 | R59022 | 0.07 | 0.08 |
| 1.55 | AG825 | 0.02 | 0.00 |
| 1.56 | 2',5'-dideoxyadenosine | 0.04 | 0.00 |
| 1.56 | Bestatin Hydrochloride | 0.02 | 0.00 |
| 1.56 | Cisplatin | 0.00 | 0.00 |
| 1.56 | Daunorubicin, HCl | 0.31 | 0.70 |
| 1.56 | L-NMMA | 0.09 | 0.00 |
| 1.56 | Lovastatin | 0.10 | 0.00 |
| 1.63 | DAPT | 0.03 | 0.00 |
| 1.63 | sunitinib malate | 0.73 | 1.11 |
| 1.63 | vorinostat | 0.03 | 0.27 |
| 1.63 | AMD3100 octahydrochloride | 0.00 | 0.00 |
| 1.63 | ATM kinase inhibitor | 0.06 | 0.00 |
| 1.63 | Ellagic acid　(Dihydrate) | 0.00 | 0.00 |
| 1.67 | 3-ATA | 0.10 | 0.00 |
| 1.67 | PD169316 | 0.11 | 0.00 |
| 1.71 | Antimycin A1 | 0.00 | 0.03 |
| 1.75 | brefeldin A | 0.00 | 0.04 |
| 1.76 | KN93 | 0.57 | 0.02 |
| 1.78 | Azacytidine | 0.17 | 0.00 |
| 1.78 | Hydroxyurea | 0.08 | 0.00 |
| 1.78 | Ro-20-1724 | 0.05 | 0.00 |
| 1.79 | Nutlin-3 | 0.16 | 0.00 |
| 1.88 | lenalidomide | 0.07 | 0.00 |
| 1.88 | PJ-34 | 0.03 | 0.00 |
| 1.89 | ML-7 | 0.26 | 0.00 |
| 1.95 | Bafilomycin A1 | 0.68 | 0.91 |
| 2.00 | Sodium salicylate | 0.04 | 0.00 |
| 2.00 | XAV939 | 0.03 | 0.00 |
| 2.01 | compound C | 0.24 | 0.00 |
| 2.13 | gefitinib | 1.06 | 1.24 |
| 2.21 | Go7874 | 0.17 | 0.90 |
| 2.22 | SB 218078 | 0.02 | 0.00 |
| 2.25 | desipramine hydrochloride | 0.04 | 0.01 |
| 2.25 | lapatinib | 0.40 | 1.12 |
| 2.50 | Jervine | 0.02 | 0.00 |
| 2.52 | ALLN | 0.03 | 0.01 |
| 2.60 | b-Rubromycin | 0.03 | 0.37 |
| 2.88 | chlorpromazine hydrochloride | 0.22 | 0.03 |
| 2.88 | cyclopamine | 0.05 | 0.00 |
| 2.88 | Akt Inhibitor VIII, Isozyme-Selective, Akti-1/2 | 0.22 | 0.00 |
| 2.88 | SU11274 | 0.62 | 0.00 |
| 2.89 | Tamoxifen, citrate | 0.31 | 1.09 |
| 3.25 | AY 9944 | 0.13 | 0.11 |
| 3.35 | Bisindolymaleimide I, HCl | 0.00 | 0.00 |
| 4.22 | NSC625987 | 0.02 | 0.00 |
